# Mechanisms Underlying the Cognitive Benefits of *Solanum macrocarpon* Leaf *n*-Butanol Extract: Acetylcholinesterase Inhibition and Oxidative Stress Modulation

**DOI:** 10.3390/plants14213283

**Published:** 2025-10-27

**Authors:** Ion Brinza, Ibukun Oluwabukola Oresanya, Ilkay Erdogan Orhan, Hasya Nazlı Gök, Lucian Hritcu, Razvan Stefan Boiangiu

**Affiliations:** 1 Faculty of Sciences, “Lucian Blaga” University of Sibiu, 7–9 Ion Ratiu Street, 550024 Sibiu, Romania; 2 Department of Pharmacognosy, Faculty of Pharmacy, Gazi University, 06330 Ankara, Türkiye; 3 Department of Pharmacognosy, Faculty of Pharmacy, Lokman Hekim University, 06510 Ankara, Türkiye; 4 Department of Biology, Faculty of Biology, Alexandru Ioan Cuza University of Iasi, 700506 Iasi, Romania

**Keywords:** neuroprotection, memory, anxiety, oxidative stress, scopolamine

## Abstract

This study investigates the neuroprotective and anxiolytic effects of *Solanum macrocarpon* L. leaf n-butanol extract (SMB) in a zebrafish model of scopolamine (SCOP; 100 μM)-induced cognitive and behavioral impairments. SCOP, a muscarinic receptor antagonist, is commonly used to mimic memory deficits and anxiety-like behaviors associated with neurodegenerative conditions. Zebrafish were chronically exposed to SMB at concentrations of 1, 3, and 6 mg/L. Behavioral assessments included anxiety-related paradigms, such as novel tank diving (NTT), novel approach (NA), and light–dark transition (LD) tests, as well as cognitive assays, including the Y-maze and novel object recognition (NOR) tests. SMB significantly mitigated SCOP-induced anxiety-like behaviors and cognitive deficits in a dose-dependent manner. Biochemical analyses demonstrated that SMB inhibited acetylcholinesterase (AChE) overactivity, indicating restoration of cholinergic function. Furthermore, SMB enhanced the activity of endogenous antioxidant enzymes, superoxide dismutase (SOD), catalase (CAT), and glutathione peroxidase (GPX) and significantly reduced oxidative stress biomarkers, including malondialdehyde (MDA) and protein carbonyls. These findings suggest that SMB may exert neuroprotective effects through modulation of cholinergic signaling and oxidative stress. Overall, SMB represents a promising phytotherapeutic candidate for mitigating cognitive and anxiety-related symptoms linked to oxidative damage. Further investigations are warranted to characterize its active constituents and assess long-term efficacy and safety in models of neurodegeneration.

## 1. Introduction

Alzheimer’s disease (AD) is a progressive neurodegenerative disorder that predominantly affects the elderly population, representing 60–70% of dementia cases worldwide [1]. Its pathology is mainly associated with the accumulation of β-amyloid (Aβ) peptides and hyperphosphorylated tau protein, leading to synaptic dysfunction, neuronal loss, and cognitive decline [2,3]. Although the precise etiology of AD remains unclear, current evidence supports a multifactorial pathogenesis involving genetic, inflammatory, metabolic, and oxidative mechanisms. The principal hypotheses—amyloid cascade, tau, inflammation, metal imbalance, and oxidative stress—highlight the complex network of processes contributing to disease progression [2,4].

According to the amyloid cascade hypothesis, the pathological accumulation of Aβ peptides-resulting from aberrant cleavage of amyloid precursor protein (APP), is an early and central event in AD. Aβ oligomers exert synaptotoxic effects and promote a range of downstream processes, including oxidative stress, mitochondrial dysfunction, and activation of neuroinflammatory cascades. In parallel, the tau hypothesis highlights the role of intracellular accumulation of hyperphosphorylated tau protein, which leads to microtubule destabilization and neurofibrillary tangle (NFT) formation. Tau pathology has been shown to correlate more strongly with cognitive decline and brain atrophy than Aβ, particularly in advanced stages of AD [5,6].

Oxidative stress plays a pivotal role in the pathogenesis of AD, resulting from excessive accumulation of reactive oxygen species (ROS) that cause DNA damage, lipid peroxidation, and protein oxidation. These ROS originate mainly from mitochondrial dysfunction, oxidative enzyme activation, and interactions between Aβ and redox-active transition metals such as iron and copper [7,8]. This imbalance accelerates Aβ and tau aggregation, contributing to synaptic dysfunction and neuronal death. In addition, aberrant neuronal cell cycle re-entry has been identified as another contributor to neurodegeneration, leading to apoptosis and tau propagation [9].

Chronic neuroinflammation represents a parallel pathological process in AD. Sustained activation of microglia and astrocytes in response to Aβ deposition triggers the release of pro-inflammatory cytokines such as IL-1β and TNF-α, which intensify oxidative stress and neuronal injury. Moreover, disruptions in metal homeostasis—particularly involving iron, copper, and zinc—further promote Aβ aggregation and oxidative damage, linking the metal and oxidative stress hypotheses [10,11,12].

A critical feature of AD is the decline in acetylcholine (ACh) levels due to enhanced acetylcholinesterase (AChE) activity, which contributes to learning and memory deficits. Clinically, AChE inhibitors such as donepezil and galantamine (GAL) are employed to mitigate cognitive decline by increasing synaptic ACh availability [2,13]. However, given the complex and interconnected nature of AD pathogenesis, current research is increasingly focused on developing multi-target therapeutic strategies aimed at simultaneously modulating Aβ and tau pathology, reducing oxidative stress, mitigating neuroinflammation, and restoring mitochondrial and synaptic function [14].

AD has a significant impact on society, with estimates suggesting that the number of patients could double by 2060 [1]. This progression imposes increasing pressure on healthcare systems and families, further aggravated by comorbidities such as viral infections, which can accelerate neurodegeneration [2]. Consequently, therapeutic approaches targeting oxidative stress and AChE dysfunction are essential to slow disease progression and improve patient outcomes [1,15].

Natural bioactive compounds, including flavonoids, polyphenols, and essential oils, have emerged as promising candidates for AD therapy due to their antioxidant, anti-inflammatory, and neuroprotective properties [16,17]. These phytochemicals can stabilize mitochondrial dynamics, enhance autophagy, and modulate key signaling pathways such as CREB activation, which supports neuronal plasticity and memory [4,18,19]. Additionally, essential oils and plant extracts have been investigated for their neuroprotective effects, demonstrating a significant impact on cognitive and behavioral functions in animal models of AD [20]. Thus, the use of plant phytocompounds as adjuncts in AD treatment offers a promising direction, aiming to halt disease progression and alleviate cognitive and behavioral symptoms.

*Solanum macrocarpon* L., also known as African eggplant, is a tropical herbaceous plant from the Solanaceae family, primarily cultivated in West Africa. Its leaves and fruits are used both in food and traditional medicine to treat conditions such as constipation, cardiovascular diseases, sore throat, and digestive issues [21,22]. The main active components include flavonoids, tannins, alkaloids, and polyphenols, which are responsible for its antioxidant and anti-inflammatory properties [23,24]. However, its use in high doses may present risks, including toxic effects [25], and caution is recommended in its administration within herbal medicine.

In vitro studies have demonstrated that the ethyl acetate and aqueous fractions of *S. macrocarpon* exhibit significant free radical scavenging capacity and inhibit oxidative enzyme activities, protecting cellular macromolecules from peroxidative damage in brain and liver tissues [26]. These extracts also show AChE and monoamine oxidase (MAO) inhibitory properties, suggesting potential application in managing neurodegenerative disorders such as AD [27,28].

Recent in silico and in vitro analyses confirm that *Solanum macrocarpon* leaf extracts act as dual-binding AChE inhibitors, supporting their role in cholinergic regulation and memory improvement [29,30]. Moreover, in in vivo models, dietary inclusion of *S. macrocarpon* alongside donepezil modulated Nrf2 redox pathways and improved memory index scores in *Drosophila* and zebrafish models of amnesia [31,32]. Similarly, studies in diabetic rat models reported attenuation of oxidative stress and improved neurotransmitter balance, indicating broader protective effects on neural and metabolic homeostasis [33]. Overall, the available evidence highlights *Solanum macrocarpon* as a rich source of neuroactive phytochemicals with promising potential in preventing oxidative, cholinergic, and inflammatory alterations associated with neurodegenerative diseases.

The zebrafish (*Danio rerio*) represents a promising experimental model for AD research due to its structural and functional similarity to the human brain and the transparency of its larvae, which allows for real-time observation of neurodegenerative processes. Scopolamine (SCOP)-induced AD models, which inhibit muscarinic acetylcholine receptors (mAChRs), induce cognitive deficits characteristic of AD, providing an efficient tool for studying the pathogenetic mechanisms of the disease [34,35]. These models allow for the analysis of neurotoxicity, oxidative stress, and behavioral alterations, facilitating the development of screening platforms for innovative therapies in the treatment of AD [36,37].

## 2. Results

### 2.1. HPLC Quantification of Phenolic and Flavonoid Derivatives

**Figure 1:**
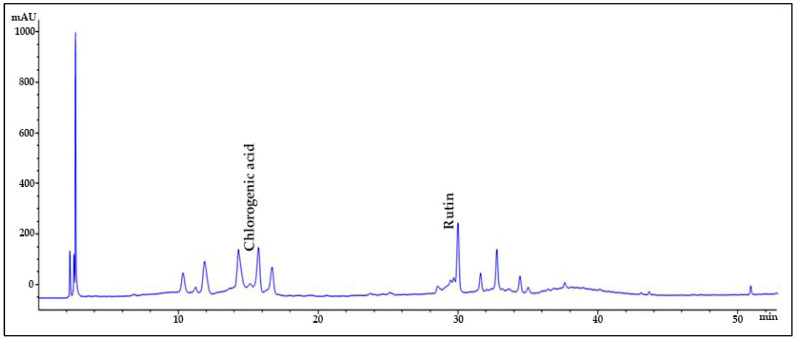
HPLC chromatogram of *Solanum macrocarpon* L. leaf *n*-butanol extract (SMB) at 260 nm.

The chromatographic analysis of phenolic compounds in *Solanum macrocarpon* L. leaf *n*-butanol extract (SMB) revealed the presence of chlorogenic acid and rutin, as presented in Figure 1, with their quantified concentrations detailed in Table 1. SMB was found to contain a high content of chlorogenic acid (162.39 ± 0.27 mg/g) and rutin (24.61 ± 0.56 mg/g), as determined from the standard calibration curve.

### 2.2. Evaluation of the Chronic Exposure Safety of Solanum macrocarpon L. leaf n-butanol extract (SMB)

The prolonged administration of the SMB at different concentrations of 1, 3, and 6 mg/L did not present any discernible indicators of toxicity in the zebrafish subjects used in the investigation, as no mortality cases were documented in any of the experimental groups. Additionally, no harmful effects were observed following treatment, indicating that SMB can be administered for extended periods without causing physical or behavioral harm to the tested organisms. These findings lend credibility to the proposal that SMB does not pose substantial toxicity risks at the concentrations mentioned and can be used in future research efforts without significant safety concerns.

### 2.3. In Silico Evaluation of the Pharmacokinetic Properties of the Main Constituents

#### 2.3.1. Physicochemical Properties of the Bioactive Compounds

The in silico evaluation of the physicochemical properties of the two main compounds identified in the analyzed SMB (chlorogenic acid and rutin) was carried out to assess their degree of drug-likeness and pharmacokinetic potential. The molecular descriptors are summarized in Table 2 and include molecular weight (MW), lipophilicity (LogP), topological polar surface area (TPSA), number of hydrogen bond acceptors and donors (HBA/HBD), compliance with Lipinski’s Rule of Five, and the QED score (quantitative estimation of drug-likeness).

Among the compounds analyzed, rutin exhibited the least favorable drug-likeness profile, characterized by a high molecular weight (610.52 Da), a large number of hydrogen bond donors (10) and acceptors (16), and an extremely high topological polar surface area (TPSA) of 269.43 Å^2^. These features are consistent with poor oral absorption and limited membrane permeability, further supported by its very low QED score (0.14) and compliance with only one of Lipinski’s four rules. Similarly, chlorogenic acid showed suboptimal drug-likeness properties, with an elevated TPSA (164.75 Å^2^) and a high number of hydrogen bond donors (6), indicating restricted permeability through biological membranes. The compound also had a relatively low QED score (0.23) and violated one of Lipinski’s rules, reinforcing its limited suitability for oral drug development.

**Figure 2:**
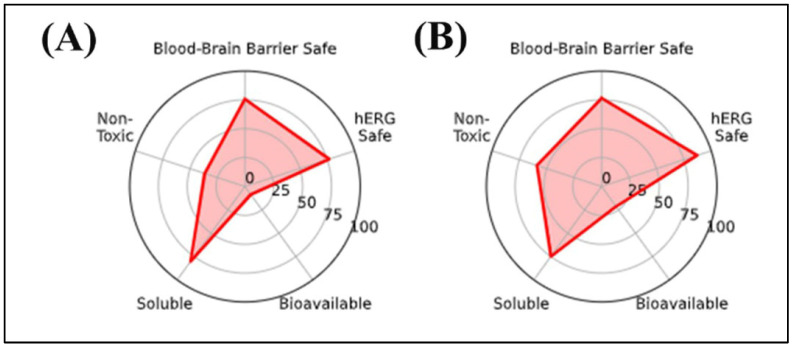
In silico radar plot analysis of key pharmacokinetic and neurotoxicity-related parameters for the selected phenolic compounds: (**A**) Chlorogenic acid and (**B**) Rutin. Parameters evaluated include blood–brain barrier permeability, hERG channel safety, general toxicity, aqueous solubility, and oral bioavailability.

The in silico radar plot analysis (Figure 2) highlighted that rutin and chlorogenic acid share favorable safety profiles, particularly regarding low predicted blood–brain barrier (BBB) penetration and negligible risk of human ether-a-go-go-related gene (hERG) channel inhibition. However, both compounds exhibited clear limitations in terms of oral bioavailability and membrane permeability, which may restrict their systemic efficacy, especially for the central nervous system (CNS) applications. Specifically, rutin’s high molecular weight and polar surface area are consistent with poor gastrointestinal absorption, while chlorogenic acid’s elevated TPSA and multiple hydrogen bond donors similarly indicate restricted permeability across biological membranes. Despite these drawbacks, their strong safety predictions support their potential as non-toxic phenolic scaffolds, although their pharmacological application would likely require formulation strategies or structural optimization to overcome absorption barriers.

Overall, the data suggest that rutin and chlorogenic acid may present limitations in terms of oral bioavailability, primarily due to their unfavorable physicochemical profiles.

#### 2.3.2. Absorption of Bioactive Compounds

To obtain a preliminary assessment of the pharmacokinetic behavior of the studied compounds, a set of in silico ADMET parameters was evaluated, with particular focus on intestinal absorption, oral bioavailability, aqueous solubility, lipophilicity, cellular permeability, and interaction with P-glycoprotein. The results of these predictions are summarized in Table 3.

Chlorogenic acid exhibited relatively high solubility (log S = –1.21) but demonstrated very low cellular permeability (logP_eff = –6.60) and only moderate intestinal absorption probability (0.88). Its negative lipophilicity (logP = –1.83) reflected a strongly hydrophilic character, consistent with reduced membrane permeability. The predicted oral bioavailability was low (0.29). In contrast, rutin displayed the weakest absorption-related pharmacokinetic profile among the compounds analyzed. It showed a very low probability of intestinal absorption (0.09), minimal predicted oral bioavailability (0.18), and the lowest cellular permeability (logP_eff = –6.82). Despite a slightly lipophilic character (logP = 0.77), rutin exhibited poor aqueous solubility (log S = –3.86), and its low P-glycoprotein inhibition probability (0.14) indicated limited ability to overcome efflux transport.

#### 2.3.3. Distribution of Bioactive Compounds

The systemic distribution of the analysed compounds was evaluated in silico and contextualized using percentile comparisons relative to reference drugs in the DrugBank database. Key pharmacokinetic parameters assessed include BBB permeability, plasma protein binding (PPB), and steady-state volume of distribution (Vdss). The results are summarized in Table 4, alongside the corresponding DrugBank percentiles, which indicate the relative positioning of each compound within the broader pharmacological distribution profile of approved drugs.

Both chlorogenic acid and rutin demonstrated low predicted BBB permeability. Rutin showed the lowest value (0.06; 3.02%), indicating very limited ability to cross the BBB, while chlorogenic acid presented a slightly higher score (0.40; 24.35%), suggesting only modest permeability under physiological conditions.

PPB values indicated moderate interaction for chlorogenic acid (59.12; 30.90%), suggesting a balanced distribution between bound and free fractions. In contrast, rutin exhibited higher tissue dispersion, with a predicted steady-state volume of distribution (Vdss) of 6.39 L/kg, consistent with extensive distribution into extravascular compartments. These parameters suggest that chlorogenic acid may maintain a relatively higher free fraction in plasma, whereas rutin is more likely to accumulate in peripheral tissues.

#### 2.3.4. Metabolism of Bioactive Compounds

The metabolic profiles of the selected compounds were assessed by estimating their probability of interaction with key enzymes from the cytochrome P450 (CYP450) family, focusing on both their potential to act as inhibitors and their likelihood of being metabolized as enzymatic substrates. In silico predictions were obtained for five major isoforms—CYP1A2, CYP2C19, CYP2C9, CYP2D6, and CYP3A4. The results, summarized in Table 5, include both predicted activity values and the corresponding percentiles relative to the DrugBank dataset, providing a comparative overview of each compound’s position within the metabolic landscape of approved drugs.

Both chlorogenic acid and rutin showed low predicted inhibition probabilities (< 0.1) across all evaluated cytochrome P450 isoforms (CYP1A2, CYP2C19, CYP2C9, CYP2D6, and CYP3A4). These values, positioned within 24–62% of the DrugBank distribution, indicate a low likelihood of enzyme-mediated drug–drug interactions and support a favorable metabolic safety profile. Substrate potential was also minimal for both compounds, with rutin showing particularly low probabilities across all isoforms, suggesting either high metabolic stability or limited hepatic biotransformation. Chlorogenic acid similarly demonstrated no notable substrate liabilities.

#### 2.3.5. Excretion of Bioactive Compounds

Excretion is a critical component of the pharmacokinetic profile, significantly influencing both the duration of action and the potential for systemic accumulation of a compound. In this study, in silico predictions focused on two key elimination parameters: plasma half-life and hepatic clearance, the latter estimated at both hepatocyte and liver microsome levels. These values provide an integrated view of each compound’s metabolic elimination potential. The results are summarized in Table 6, along with the corresponding percentiles from the DrugBank dataset, offering a comparative reference to approved drugs.

Rutin was the only compound predicted to exhibit a prolonged plasma half-life (49.51 h; 87.05%), suggesting slow elimination and a potential for systemic accumulation during repeated dosing. This extended retention was consistent with moderate hepatic microsomal clearance (40.10 µL/min/mg) and relatively low hepatocellular clearance (25.57 µL/min/10^6^ cells), indicating limited metabolic turnover. In contrast, chlorogenic acid showed a very short, near-zero predicted half-life (0.00 h) and moderate hepatocellular clearance (22.94 µL/min/10^6^ cells), coupled with negligible microsomal clearance, suggesting rapid elimination, possibly via renal excretion or non-enzymatic pathways rather than extensive hepatic metabolism.

#### 2.3.6. Toxicity of Bioactive Compounds

Table 7 summarizes the predicted toxicity parameters of the analysed compounds, based on in silico modelling, and provides insights into their potential safety profiles. The evaluated parameters include hERG channel blockade, clinical toxicity, mutagenicity, drug-induced liver injury (DILI), carcinogenicity, acute toxicity (LD_50_), skin sensitization, and interactions with various nuclear receptors and molecular targets relevant to toxicity and endocrine disruption.

Regarding hERG channel inhibition, rutin showed a higher predicted probability (0.65; 69.99%), indicating an increased risk of cardiotoxicity, whereas chlorogenic acid presented a much lower score (0.06; 23.26%). Predicted clinical toxicity was moderate for both compounds, with scores of 0.20 (65.99%) for rutin and 0.17 (63.28%) for chlorogenic acid. Mutagenicity was elevated for rutin (0.60; 88.02%), suggesting a greater genotoxic risk, while chlorogenic acid was not flagged as having notable mutagenic potential. Both compounds displayed moderate drug-induced liver injury (DILI) probabilities (0.75; 67.74% for rutin and 0.55; 59.21% for chlorogenic acid). Predicted carcinogenicity remained low for both compounds (< 0.15). In terms of acute toxicity, rutin showed a lower predicted toxicity (LD_50_ = 2.90; 73.40%) compared to chlorogenic acid (2.11; 45.87%). Regarding skin sensitization, chlorogenic acid (0.28; 32.11%) and rutin (0.21; 22.64%) both showed low dermatological risk.

Overall, the data suggest that while chlorogenic acid presents a relatively safer toxicological profile, rutin exhibits higher probabilities of cardiotoxicity and mutagenicity, underscoring the need for cautious evaluation in further studies.

#### 2.3.7. PASS Predictions of Neuropharmacological Activities and Safety Profile of Phenolic Compounds

The neuropharmacological profiles of chlorogenic acid and rutin were comprehensively evaluated using PASS predictions (Table 8). All compounds demonstrated high probabilities for antioxidant activity, with rutin (Pa = 0.923, Pi = 0.003) and chlorogenic acid (Pa = 0.785, Pi = 0.004) standing out, highlighting their neuroprotective potential through oxidative stress reduction. Similarly, all compounds showed favorable scores as oxidoreductase inhibitors (Pa > 0.64), supporting their role in modulating oxidative enzymes.

PASS predictions indicated that both rutin and chlorogenic acid exhibited high probabilities of antioxidant activity (Pa = 0.923 and 0.785, respectively), supporting their potential neuroprotective role through oxidative stress reduction. Both compounds also scored strongly as lipid peroxidase inhibitors, with rutin showing an exceptionally high probability (Pa = 0.987, Pi = 0.001) and chlorogenic acid also demonstrating notable activity (Pa = 0.855, Pi = 0.003). In contrast, their predicted activities in G-protein-coupled receptor modulation were comparatively lower (Pa < 0.72).

With respect to dementia therapy, rutin showed a moderate predicted probability (Pa = 0.541, Pi = 0.008), whereas chlorogenic acid had the lowest score among the analyzed compounds (Pa = 0.258). Importantly, both compounds displayed high probabilities for neurotoxicity (Pa = 0.882 for rutin; Pa = 0.874 for chlorogenic acid), suggesting potential safety concerns in neurological applications.

### 2.4. Effects on Anxiety-like Behavior in the NTT

**Figure 3:**
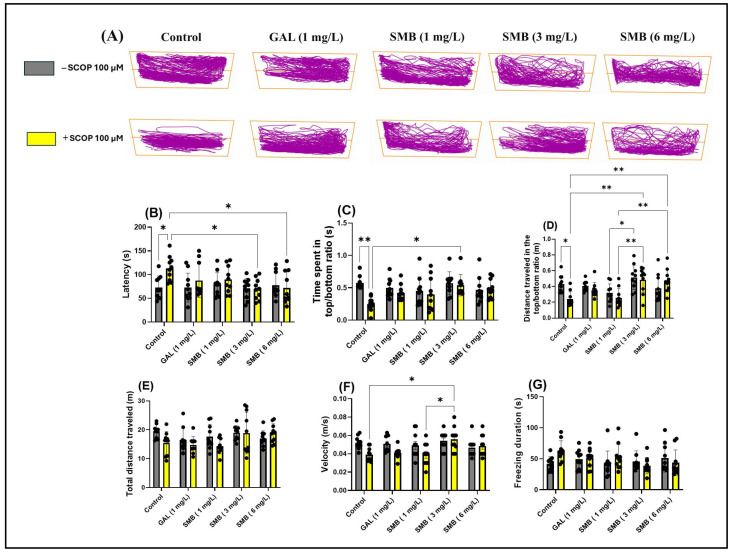
The effect of the *Solanum macrocarpon* L. leaf *n*-butanol extract (SMB) administered chronically at concentrations of 1, 3, and 6 mg/L, on native zebrafish and those additionally acutely treated with scopolamine (SCOP, 100 µM) in the novel tank diving test (NTT). Galantamine (GAL, 1 mg/L) was used as a positive control. (**A**) Graphical representation of the swimming trajectories of zebrafish during the NTT; (**B**) Latency (s), (**C**) Ratio of time spent in the top versus bottom zone (s), (**D**) Ratio of the distance traveled in the top versus bottom zone, (**E**) Total distance traveled (m), (**F**) Velocity (m/s), and (**G**) Freezing duration (s). The results are presented as mean values ± S.E.M. (n = 10). Statistical significance based on Tukey’s post hoc test: * *p* < 0.05 and ** *p* < 0.01.

The representative swimming behavior trajectories observed in zebrafish during the NTT (Figure 3A) highlighted a pronounced level of anxiety in the SCOP-induced cohort, reflected by a marked tendency to explore the bottom area of the tank compared to the control cohort. In contrast, the groups treated with SMB demonstrated an increased level of spatial exploration, suggesting a potential anxiolytic effect.

Two-way ANOVA analysis revealed significant changes associated with chronic SMB treatment regarding anxiety parameters, including latency to explore the top zone [F(1, 90) = 4.592, *p* = 0.0348] (Figure 3B), ratio of time spent in the top/bottom tank zones [F(1, 90) = 7.133, *p* < 0.0001] (Figure 3F), ratio of distance traveled in the top /bottom tank zones [F(1, 90) = 14.78, *p* = 0.0090] (Figure 3C), and ratio of distance travelled in both zones [F(1, 90) = 3.446, *p* = 0.0667] (Figure 3D). In contrast, two-way ANOVA analysis did not show significant statistical changes regarding the total distance traveled by zebrafish during the NTT across the two cohorts [F(1, 90) = 3.755, *p* = 0.0558] (Figure 3E); however, significant changes were observed in locomotor activity parameters, such as velocity [F(1, 90) = 19.27, *p* = 0.0046] (Figure 3F), while freezing duration [F(1, 90) = 1.255, *p* = 0.2656] (Figure 3G) showed no significant changes.

Post hoc Tukey test revealed a reduction in anxiety-like behavior in zebrafish treated with SCOP, indicated by decreased latency at concentrations of 3 and 6 mg/L (*p* < 0.05) (Figure 3B). Simultaneously, at the middle concentration, chronic SMB treatment also induced an increase in exploration of the top zone (*p* < 0.05) (Figure 3C). In terms of the ratio of distance traveled in the top/bottom zones, both the 3 mg/L and 6 mg/L concentrations were effective in restoring this ratio (*p* < 0.01) (Figure 3D). Additionally, significant differences were observed between groups treated with SMB at 3 mg/L and those treated with SMB at 1 mg/L (*p* < 0.05) (Figure 3D). Similarly, differences were noted between groups treated with SMB + SCOP at 3, 6 mg/L and those treated with SMB + SCOP at 1 mg/L (*p* < 0.01) (Figure 3D).

Regarding total distance traveled and freezing duration, no significant differences were observed between the experimental groups (Figure 3E,G), suggesting that SMB does not modify the general locomotor activity of zebrafish at the concentrations used. However, a slight increase in velocity was observed in the groups treated with SMB + SCOP at 3 mg/L compared to the SCOP group (*p* < 0.05) and compared to SMB + SCOP at 1 mg/L (*p* < 0.05) (Figure 3F). GAL (1 mg/L) was used as a reference pharmacological agent in the NTT to compare with the effects of SMB treatments.

### 2.5. Effects on Anxiety-like Behavior in the NAT

**Figure 4:**
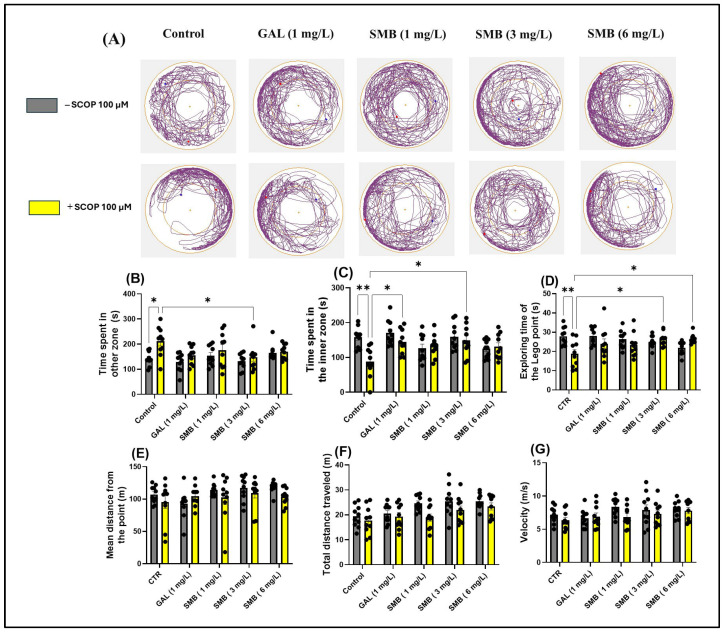
The effect of the *Solanum macrocarpon* L. leaf *n*-butanol extract (SMB) treatment, given at concentrations of 1, 3, and 6 mg/L, on both native zebrafish and those subjected to scopolamine (SCOP, 100 µM) in the novel approach test (NAT). Galantamine (GAL, 1 mg/L) served as a positive control. (**A**) Graphical representation of fish swimming paths during the NAT, central Lego reference point (+); blue dot - starting position of the fish at the beginning of the test; red dot - the end point of the fish’s trajectory at the end of the observation period; (**B**) Time spent in other zone (s); (**C**) Time spent in inner zone (s); (**D**) Exploring time of the Lego point (s); (**E**) Mean distance from the Lego point (m); (**F**) Total distance travelled (m); (**G**) velocity (s). The results are presented as mean values ± S.E.M. (n = 10 animals per group). Statistical significance based on Tukey’s post hoc test: * *p* < 0.05 and ** *p* < 0.01.

Representative swimming trajectories of zebrafish in the NAT (Figure 4A) showed that, compared to the control group, the SCOP-treated group experienced higher anxiety. This was demonstrated by the predominant exploration of the outer zone of the tank. Two-way ANOVA showed that zebrafish experienced significant changes in parameters assessing anxiety-like behavior, such as time spent in the outer zone [F(1, 90) = 10.30, *p* = 0.0018] (Figure 4B), time spent in the inner zone [F(1, 90) = 6.857, *p* = 0.0104] (Figure 4C), exploring time of the Lego point [F(1, 90) = 4.255, *p* = 0.0420] (Figure 4D), and mean distance from the Lego point [F(1, 90) = 2.680, *p* = 0.1051] (Figure 4E). Additionally, two-way ANOVA was used to evaluate the effectiveness of chronic SMB treatment on the locomotor activity of zebrafish, such as total distance traveled [F(1, 90) = 9.290, *p* = 0.0030] (Figure 4E) and velocity [F(1, 90) = 3.180, *p* = 0.0779] (Figure 4F).

According to post hoc analyses performed using the Tukey test, daily exposure to chronic SMB treatment had a significant impact on anxiety-related behaviors in the NAT in zebrafish. In the SMB + SCOP groups, SMB treatment reduced the time spent in the outer zone, exclusively at the middle concentration (*p* < 0.05) (Figure 4B), while increasing the time spent in the inner zone (3 mg/L, *p* < 0.05) (Figure 4C). GAL (1 mg/L), which served as the reference drug in the NAT, also indicated a significant increase in time spent in the inner zone (*p* < 0.05) (Figure 4C). Simultaneously, in the SMB + SCOP groups, SMB treatment intensified the exploring time of the Lego point, but only at concentrations of 3 and 6 mg/L (*p* < 0.05) (Figure 4D). Post hoc Tukey analyses, however, did not detect any effect of the treatment on the mean distance from the Lego point (Figure 4E). Furthermore, SMB treatment did not modify the locomotor activity of zebrafish in the NAT (Figure 4F,G). An increase in hyperlocomotion behavior because of acute SCOP treatment was observed (*p* < 0.05) (Figure 4B).

### 2.6. Effects on Anxiety-like Behavior in LDT

**Figure 5:**
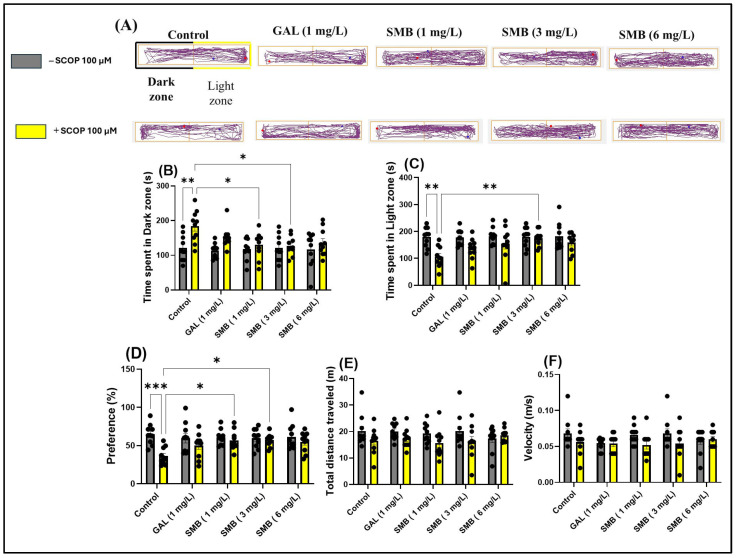
The impact of treatment with the *Solanum macrocarpon* L. leaf *n*-butanol extract (SMB), administered at concentrations of 1, 3, and 6 mg/L, on native zebrafish and those treated with scopolamine (SCOP, 100 µM) in the light/dark test (LDT). Galantamine (GAL, 1 mg/L) served as a positive control. (**A**) Graphical representation of the swimming trajectories of zebrafish during the LDT, blue dot—the starting point of the fish at the beginning of the test, red dot—the ending point of the trajectory at the end of the test period; (**B**) Time spent in the Light zone (s); (**C**) Time spent in the dark zone (s); (**D**) Preference (%); (**E**) Total distance traveled (m) and (**F**) Velocity (m/s). Results are presented as mean ± S.E.M. (n = 10). Statistical significance based on Tukey’s post hoc test: * *p* < 0.05, ** *p* < 0.01, and *** *p* < 0.001.

In the LDT, representative swimming trajectories of zebrafish (Figure 5A) revealed an increased level of anxiety in the SCOP-treated group, characterized by a predominant exploration of the dark zone of the aquarium compared to the control group. Two-way ANOVA analysis of the LDT revealed significant effects of SMB treatment on anxiety parameters, such as: time spent in the dark zone [F(1, 90) = 14.50, *p* = 0.0003] (Figure 5D), time spent in the light zone [F(1, 89) = 17.66, *p* < 0.0001] (Figure 5E), and preference [F(1, 90) = 13.85, *p* = 0.0003] (Figure 5F). However, two-way ANOVA did not indicate significant changes in the locomotor activity of zebrafish, regardless of cohort or group, such as total distance travelled [F(1, 90) = 7.106, *p* = 0.0091] (Figure 5B) and velocity [F(1, 90) = 5.666, *p* = 0.0194] (Figure 5C).

Post hoc analyses using the Tukey test demonstrated that acute treatment with SCOP and SMB at lower concentrations (1 and 3 mg/L) decreased the time spent in the dark zone (*p* < 0.05) (Figure 5B) and zebrafish preference for the light zone (*p* < 0.05) (Figure 5D), compared to zebrafish treated exclusively with SCOP. Additionally, in the SMB + SCOP group, at a concentration of 3 mg/L, there was a significant increase in time spent in the light zone (*p* < 0.01) (Figure 5C), compared to the group that received only SCOP. These results highlight the potential anxiolytic effect of SMB and suggest a possible influence on neurobehavioral mechanisms associated with stress and anxiety.

### 2.7. Effects on Spatial Memory in Y-Maze

**Figure 6:**
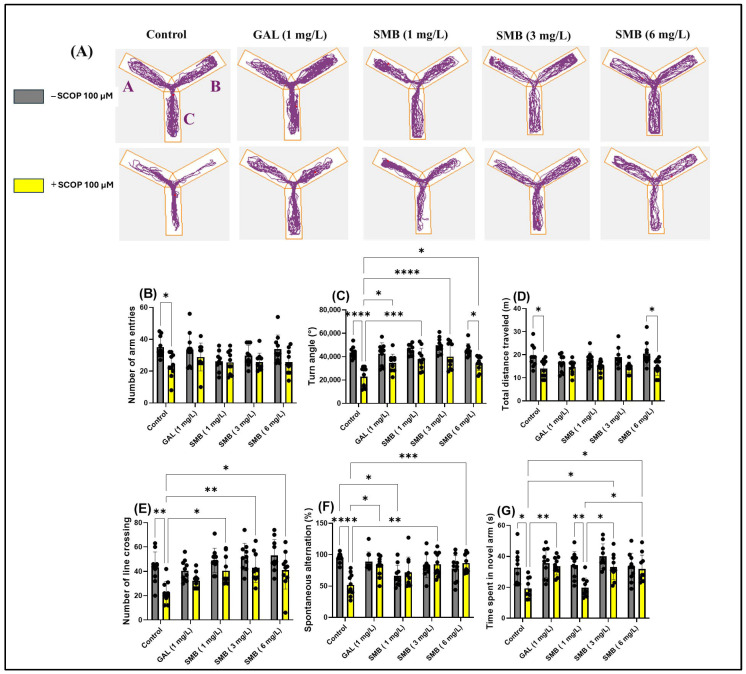
The impact of *Solanum macrocarpon* L. leaf *n*-butanol extract (SMB), administered at concentrations of 1, 3, and 6 mg/L, on native zebrafish and those treated with scopolamine (SCOP, 100 µM) in the Y-maze. Galantamine (GAL, 1 mg/L) served as a positive control. (**A**) Graphical representation of the swimming trajectories of zebrafish during the Y-maze test; (**B**) Number of line entries; (**C**) Turn angle (°); (**D**) Total distance travelled (m); (**E**) Number of line crossings; (**F**) Spontaneous alternation (%); (**G**) Time spent in the novel arm (s). The results are presented as mean values ± S.E.M. (n = 10). Statistical significance based on Tukey’s post hoc test: * *p* < 0.05, ** *p* < 0.01, *** *p* < 0.001, and **** *p* < 0.0001.

The use of the Y-maze allowed the evaluation of the effects of SMB administration on the tendency of zebrafish to explore novel environments and, specifically, on spatial memory. Monitoring the trajectories of zebrafish treated with SCOP highlighted significant deficits in exploratory response to unfamiliar environments, manifested by a reduction in activity in the new arm of the maze (Figure 6A). In contrast, chronic administration of SMB and acute GAL administration led to a significant increase in activity in this arm, suggesting a beneficial effect on exploratory behavior.

The two-way ANOVA analysis revealed significant changes in the locomotor activity of zebrafish, including notable effects on the number of entries into arms [F(1, 90) = 14.72, *p* < 0.0002] (Figure 6B), the turning angle [F(1, 90) = 56.62, *p* < 0.0001] (Figure 6C), and the total distance traveled [F(1, 90) = 32.48, *p* < 0.0001] (Figure 6D). Additionally, the treatment significantly influenced spatial memory, highlighted by changes in the number of line crossings [F(1, 90) = 26.72, *p* < 0.0001] (Figure 6E), spontaneous alternation [F(1, 90) = 3.620, *p* = 0.0603] (Figure 6F), and the time spent in the novel arm [F(1, 90) = 20.98, *p* < 0.0001] (Figure 6G).

Post hoc analyses using the Tukey test indicated that SCOP treatment significantly reduced the number of entries into arms (*p* < 0.05) (Figure 6B), the turning angle (*p* < 0.0001) (Figure 6C), the total distance traveled (*p* < 0.05) (Figure 6D), the number of line crossings (*p* < 0.01) (Figure 6E), spontaneous alternation (*p* < 0.0001) (Figure 6F), and the time spent in the novel arm (*p* < 0.05) (Figure 6G), compared to the control group. On the other hand, zebrafish chronically treated with SMB + SCOP (100 µM) demonstrated improved locomotor activity, reflected by an increase in the turn angle at concentrations of 1 mg/L (*p* < 0.01), 3 mg/L (*p* < 0.0001), and 6 mg/L (*p* < 0.05) (Figure 6C), similar to GAL (*p* < 0.05) (Figure 6C). Additionally, a positive correlation was found regarding the turn angle between fish treated with SMB 6 mg/L vs. fish in the SMB 6 mg/L + SCOP group (*p* < 0.05) (Figure 6C). At the same time, SMB improved spatial memory by increasing the number of line crossings at concentrations of 1 and 6 mg/L (*p* < 0.05) and especially at 3 mg/L (*p* < 0.01) (Figure 6E), spontaneous alternation at 3 and 6 mg/L (*p* < 0.001 and *p* < 0.01, respectively) (Figure 6F). Simultaneously, the SCOP + GAL group showed an improvement in spontaneous alternation (*p* < 0.05) compared to the SCOP-only group (Figure 6F). Similarly, post hoc analyses using the Tukey test demonstrated an improvement in memory in zebrafish following exposure to SMB, by increasing the time spent in the novel arm at 3–6 mg/L (*p* < 0.05) (Figure 6G). Additionally, an increase in exploration time in the novel arm was observed in the groups treated with SCOP + GAL vs. SCOP (*p* < 0.01) (Figure 6G), SCOP + SMB (3–6 mg/L) vs. SCOP + SMB (1 mg/L) (*p* < 0.05) (Figure 6G), and SMB (1 mg/L) vs. SCOP + SMB (1 mg/L) (*p* < 0.05) (Figure 6G). These findings support the hypothesis that SMB can enhance exploration and spatial memory, with a neuroprotective potential comparable to that of GAL.

### 2.8. Effects on Recognition Memory in NOR

**Figure 7:**
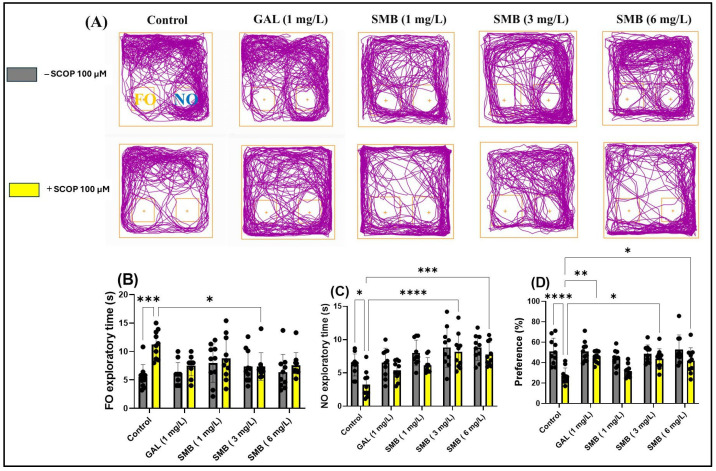
The impact *Solanum macrocarpon* L. leaf *n*-butanol extract (SMB) treatment, administered at concentrations of 1, 3 and 6 mg/L, on native zebrafish and those treated with scopolamine (SCOP, 100 µM) in the novel object recognition test (NOR). Galantamine (GAL, 1 mg/L) served as a positive control. (**A**) Graphical depiction of zebrafish swimming paths during the NOR; (**B**) Familiar object (FO) exploratory time (s); (**C**) Novel object (NO) exploratory time (s), and (**D**) Preference (%). The results are presented as mean values ± S.E.M. (n = 10). Statistical significance based on Tukey’s post hoc test: * *p* < 0.05, ** *p* < 0.01, *** *p* < 0.001, and **** *p* < 0.0001.

The analysis of the representative trajectories of zebrafish treated with SCOP revealed significant deficits in recognition memory, reflected by reduced exploration of the novel object (NO) compared to the familiar object (FO). However, the administration of SMB and GAL resulted in a significant increase in preference for NO in the NOR test (Figure 7A). The two-way ANOVA analysis highlighted significant effects of the treatment on recognition memory, manifested by significant differences in the time spent exploring the FO [F(1, 90) = 11.63, *p* = 0.0010] (Figure 7B), the NO [F(1, 90) = 14.37, *p* = 0.0003] (Figure 7C), and preference percentage for NO [F(1, 90) = 32.93, *p* < 0.0001] (Figure 7D).

Post hoc Tukey test showed that treatment with SCOP significantly increased the time spent exploring FO (*p* < 0.001) (Figure 7B), while simultaneously reducing the time spent exploring NO (*p* < 0.05) (Figure 7C) and preference percentage for NO (*p* < 0.0001) (Figure 7D). In contrast, chronic treatment with SMB administered daily to zebrafish had a significant effect on the time spent exploring FO, only at the 3 mg/L concentration (*p* < 0.05) (Figure 7B). However, regarding the exploration time of NO, both the 3 mg/L (*p* < 0.001) (Figure 7C) and 6 mg/L (*p* < 0.001) (Figure 7C) concentrations proved to be effective, along with a significant increase in preference percentage for NO, in a similar manner for both previously mentioned concentrations (*p* < 0.05) (Figure 7D). Additionally, GAL (1 mg/L), used as a reference in the NOR test, increased preference percentage for NO (*p* < 0.01) (Figure 7D).

Thus, for the first time, we have elucidated the specific effects of SMB treatment on recognition memory and the behavior of exploring novel objects in zebrafish. These findings underscore the potential of SMB treatment in enhancing memory and recognition behavior.

### 2.9. Effects on Brain AChE Activity

**Figure 8:**
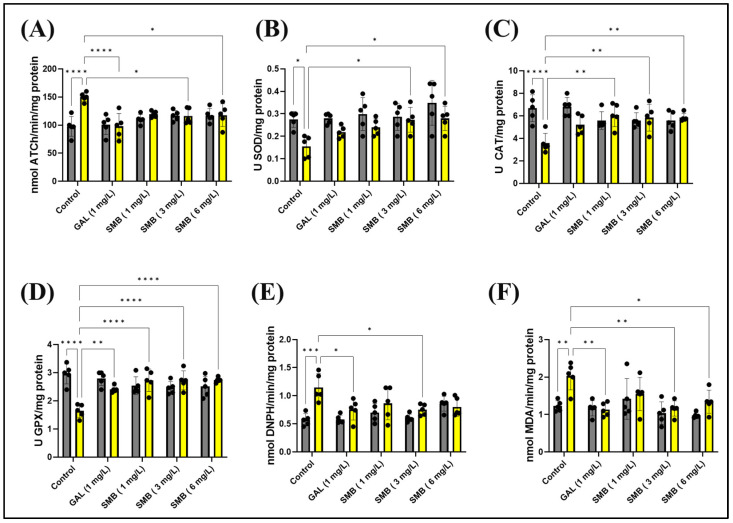
The treatment with the *Solanum macrocarpon* L. leaf *n*-butanol extract (SMB) at concentrations of 1, 3, and 6 mg/L on native zebrafish and those treated with scopolamine (SCOP, 100 µM) affected the following biological parameters: (**A**) Acetylcholinesterase (AChE) activity; (**B**) Superoxide dismutase (SOD) activity; (**C**) Catalase (CAT) specific activity; (**D**) Glutathione peroxidase (GPX) activity; (**E**) Carbonylated protein levels, and (**F**) Malondialdehyde (MDA) content. The results are presented as mean values ± S.E.M. (n = 5). According to Tukey’s post hoc test, statistical significance is as follows: * *p* < 0.05, ** *p* < 0.01, *** *p* < 0.001, and **** *p* < 0.0001.

The enzymatic activity of AChE in the zebrafish brain was meticulously investigated to elucidate the mechanisms by which SMB mitigates memory deficits induced by acute exposure to SCOP at a concentration of 100 μM. A statistical evaluation using two-way ANOVA indicated a significant influence of the treatment on AChE activity [F(1, 40) = 8.138, *p* = 0.0068], as shown in Figure 8A. Acute administration of SCOP before euthanasia led to a substantial increase in AChE activity in the zebrafish brain when compared to the control cohort (*p* < 0.0001), suggesting cholinergic dysfunction associated with cognitive impairment. In contrast, SMB administration resulted in a notable decrease in enzymatic activity, with a particularly pronounced effect at concentrations of 3 and 6 mg/L (*p* < 0.05) when compared to the group treated exclusively with SCOP (Figure 8A). GAL, at a concentration of 1 mg/L, also reduced AChE activity in the brain of zebrafish exposed to acute SCOP action (*p* < 0.0001) (Figure 8A).

These findings highlight the therapeutic promise of SMB in restoring cholinergic system balance, thus reinforcing the hypothesis of a neuroprotective mechanism in attenuating cognitive impairment induced by SCOP.

### 2.10. Effects on Brain Oxidative Status

To further explore the mechanisms through which SMB exerts protective effects against memory impairment, its influence on key markers of oxidative stress and redox balance in the brain of zebrafish was investigated. Given the well-established connection between oxidative stress and cognitive dysfunction, the study focused on the relationship between ROS generation and antioxidant defense mechanisms. In this regard, the activity of essential antioxidant enzymes, including SOD, CAT, and GPX, was assessed to determine SMB’s capacity to counteract oxidative stress. Simultaneously, markers of oxidative damage, such as protein oxidation (carbonylated protein concentration) and lipid peroxidation (MDA levels), were quantified to estimate the extent of SMB’s protective effects. Zebrafish brain tissue was used for these analyses, given the validity of this model in neurobiological and cognitive research.

Statistical analysis using two-way ANOVA indicated a significant effect of treatment on all parameters related to oxidative stress and the antioxidant system. Specifically, the treatment significantly influenced SOD activity [F(1, 40) = 16.58, *p* = 0.0002] (Figure 8B), CAT activity [F(1, 40) = 8.630, *p* = 0.0055] (Figure 8C), GPX activity [F(1, 40) = 6.254, *p* = 0.0166] (Figure 8D), carbonylated protein concentration [F(1, 40) = 17.66, *p* = 0.0001] (Figure 8E), and MDA levels [F(1, 40) = 9.934, *p* < 0.0031] (Figure 8F). These results suggest that SMB modulates the redox balance in the zebrafish brain and contributes to reducing oxidative damage, providing a plausible mechanism for its memory-enhancing effects.

Post hoc analysis using the Tukey test provided additional insights into the specific effects of treatments on oxidative stress markers. SCOP administration clearly had a negative impact on the zebrafish antioxidant system. Specifically, SCOP caused a significant decrease in SOD activity (*p* < 0.05) (Figure 8B), CAT activity (*p* < 0.0001) (Figure 8C), and GPX activity (*p* < 0.0001) (Figure 8D). Concurrently, SCOP increased markers of oxidative damage, including carbonylated protein concentration (*p* < 0.001) (Figure 8E) and MDA levels (*p* < 0.001) (Figure 8F). These results confirm that SCOP induces oxidative stress in the brain, which may contribute to its negative effects on memory.

On the other hand, SMB treatment demonstrated the ability to counteract the harmful effects of SCOP on the antioxidant system. SMB administration led to an increase in SOD activity at concentrations of 3 and 6 mg/L (*p* < 0.05) (Figure 8B). SMB also improved CAT activity at all three concentrations tested (*p* < 0.001) (Figure 8C). Similarly, SMB enhanced GPX activity at all three concentrations (*p* < 0.0001), in a manner similar to GAL (*p* < 0.01) (Figure 8D). Additionally, SMB at a concentration of 3 mg/L reduced carbonylated protein concentration in a manner similar to GAL (*p* < 0.05) (Figure 8E). Furthermore, SMB also reduced MDA levels (3 mg/L, *p* < 0.01 and 6 mg/L, *p* < 0.05) (Figure 8F). GAL also had a significant effect on MDA levels (*p* < 0.01) (Figure 8F). These results clearly suggest that SMB possesses antioxidant properties and can alleviate oxidative stress induced by SCOP. The increase in antioxidant enzyme activity, coupled with the reduction of oxidative damage markers, supports the hypothesis that SMB exerts neuroprotective effects and memory-enhancing properties. Furthermore, the dose-dependent nature of these effects reinforces the link between SMB’s antioxidant activity and its potential therapeutic benefits.

### 2.11. Pearson Correlations Between Behavioral and Biochemical Variables

**Figure 9:**
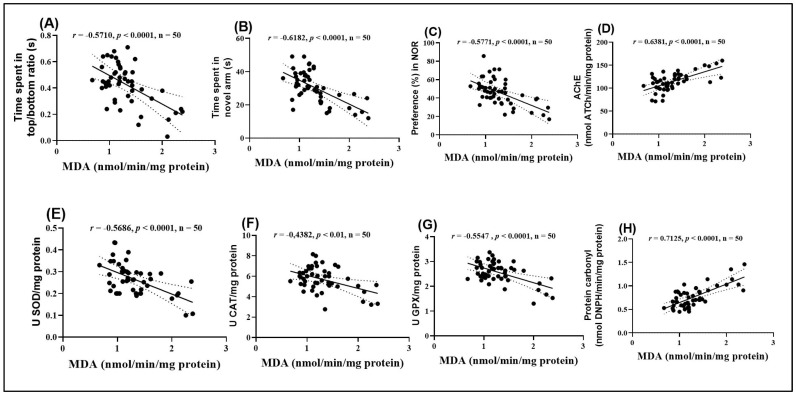
Correlation analyses between behavioral and biochemical parameters (Pearson correlation). Data presented are: (**A**) time spent by fish in the top/bottom zone of the NTT test compared to MDA levels (n = 50, *r* = −0.5710, *p* < 0.0001); (**B**) time spent by fish in the novel arm of the Y-maze compared to MDA (n = 50, *r* = −0.6182, *p* < 0.0001); (**C**) percentage preference in the NOR test compared to MDA (n = 50, *r* = −0.5771, *p* < 0.0001); (**D**) AChE activity compared to MDA (n = 50, *r* = 0.6381, *p* < 0.0001); (**E**) SOD activity compared to MDA (n = 50, *r* = −0.5686, *p* < 0.0001); (**F**) CAT activity compared to MDA (n = 50, *r* = −0.4382, *p* < 0.01); (**G**) GPX activity compared to MDA (n = 50, *r* = −0.5547, *p* < 0.0001), and (**H**) carbonylated protein levels compared to MDA (n = 50, *r* = 0.7125, *p* < 0.0001).

To further investigate the relationships between observed behavioral changes, enzymatic activities, and oxidative stress, a Pearson correlation analysis was performed. This analysis aimed to determine the intensity and direction of the linear relationships between several essential parameters. Among these, the parameters included the time spent in the top and bottom zones of the tank (NTT), the time spent exploring the novel arm in the Y maze, the preference percentage (NOR), AChE, SOD, CAT, and GPX activities, as well as carbonylated protein and MDA levels. The results of this analysis are presented in Figure 9.

The analysis highlighted significant negative correlations between MDA levels and several behavioral and enzymatic parameters. Specifically, the time spent in the top/bottom zones in the NTT (Figure 9A), the time spent exploring the novel arm in the Y maze (Figure 9B), the preference percentage in the NOR (Figure 9C), and the activities of SOD (Figure 9E), CAT (Figure 9F), and GPX (Figure 9G) all showed significant negative correlations with MDA (r = −0.5710, −0.6182, −0.7017, −0.5771, −0.5686, −0.4382, and −0.5547, respectively). These negative correlations suggest that high levels of MDA, indicative of enhanced lipid peroxidation and oxidative stress, are associated with impaired behavioral performance and reduced antioxidant enzyme activity.

On the other hand, MDA levels exhibited significant positive correlations with AChE activity (Figure 9D) and carbonylated protein concentration (Figure 9H). The correlation between MDA and AChE activity was particularly strong (r = 0.6381), suggesting a close relationship between oxidative stress and dysfunction of the cholinergic system. Additionally, the positive correlation between MDA and carbonylated proteins (r = 0.7127) supports the hypothesis that oxidative stress contributes to protein damage. These results provide further evidence supporting the hypothesis that oxidative stress plays a central role in the behavioral and neurochemical changes observed.

## 3. Discussion

To evaluate the possibility of ameliorating the behavioral deficits induced by SCOP, three concentrations of SMB (1, 3, and 6 mg/L) were administered long-term to zebrafish treated with SCOP. The study analyzed behavioral effects using well-known in vivo tests, such as anxiety tests (NTT, NAT, and LDT) as well as memory tests (Y-maze and NOR). The results showed that SMB treatment had a significant impact on reducing the SCOP-induced anxious behavior in the NTT, NAT, and LDT and improved the affected memory, as observed in the Y-maze and NOR tests. These findings are supported by the existing literature and align with the results of our previous studies, which revealed that exposure to SCOP generates variable behavioral changes, predominantly influenced by the dosage and duration of treatment [39,40]. These results are consistent with those observed by other research teams, indicating that SCOP exposure in zebrafish leads to significant cognitive deficits and anxiety-like behaviors. These effects are evident in behavioral assessments, such as the NTT and LDT, where an increase in anxiety is observed [41]. Furthermore, memory deficits are evident in tasks such as the Y-maze, highlighting the impact of SCOP on neurotransmission and its relevance in modeling cognitive disorders, such as AD [42,43]. Regarding *Solanum macrocarpon*, also known as African eggplant, it has demonstrated beneficial effects on mental health. The extracts from its leaves have shown anxiolytic and neuroprotective potential in rodent studies, reducing anxiety-like behaviors and improving cognitive functions.

Although in silico predictions indicated limited BBB permeability for both chlorogenic acid and rutin (Table 4), this observation does not contradict the neuroprotective efficacy seen in vivo. These phenolic compounds can exert indirect systemic effects by reducing peripheral oxidative stress and inflammatory signaling, which secondarily benefits neuronal function. Moreover, both chlorogenic acid and rutin are bio-transformed into smaller, more lipophilic metabolites such as caffeic and ferulic acids, which display significantly higher BBB permeability and established neuroactivity [44].

Although the PASS algorithm predicted a relatively high probability of neurotoxicity for rutin (Pa = 0.87–0.88), such computational results should be interpreted with caution. These models rely mainly on structural similarity and fail to account for dose-dependent hormetic behavior, metabolism, or the antioxidant–pro-oxidant balance typical of polyphenols. Recent studies indicate that rutin exhibits a biphasic, hormetic dose–response pattern, acting as an antioxidant and neuroprotective compound at physiological concentrations while only exerting pro-oxidant activity at high, non-physiological doses [45].

Experimental evidence supports this interpretation: rutin and its glycosides have been shown to protect neuronal cells against 6-hydroxydopamine-induced damage by enhancing endogenous antioxidant defenses and suppressing lipid peroxidation [46]. Similarly, in vivo and preclinical models demonstrated that rutin mitigates xenobiotic-induced oxidative stress and neuroinflammation, providing neuroprotection through modulation of the Nrf2/ARE pathway [47,48].

Therefore, the apparent “neurotoxicity” predicted in silico most likely reflects the redox reactivity underlying hormetic adaptation rather than genuine toxicity. This interpretation is consistent with recent in vivo evidence showing that polyphenolic-rich extracts containing rutin improve cognitive function and antioxidant status in SCOP exposed zebrafish [39,49]. To validate these computational predictions, future studies should include in vitro assays assessing cell viability, oxidative stress markers, and concentration-dependent effects of rutin. Such investigations will clarify whether the predicted neurotoxicity corresponds to physiological pro-oxidant signaling or actual cytotoxicity.

Additionally, SMB represents a complex phytochemical matrix, and synergistic interactions among minor constituents (e.g., flavonoids, saponins, alkaloids) may enhance transport or facilitate adaptive responses that mimic central nervous system modulation [50]. From a mechanistic standpoint, the principle of neurohormesis offers a coherent framework for explaining this apparent paradox. Neurohormesis describes the phenomenon by which low, non-toxic doses of stress-inducing phytochemicals activate cellular defense pathways—such as Nrf2, HO-1, and HSP70—that bolster neuronal resilience [45,51]. Polyphenols like chlorogenic acid and rutin can act as mild electrophilic stimuli, eliciting adaptive redox signaling rather than direct antioxidation. This hormetic activation strengthens antioxidant defenses, improves mitochondrial function, and enhances neuronal survival even when direct CNS penetration is limited [40,49].

In one study, administration of this extract decreased lipid peroxidation and improved neurotransmission in animals with paracetamol-induced brain injuries [52]. Ogunsuyi et al. [53] also reported that pretreatment with dietary inclusion of the leaf extract of *S. macrocarpon* significantly reversed the impairment in the rat spatial working memory induced by SCOP. In a similar way, increases in the activity of AChE, BChE and monoamine oxidase caused by SCOP were significantly reversed in rats that had been given dietary additions of this extract. What is more, the effect of SCOP on impaired antioxidant status could be counteracted by the prior administration of dietary additions of *S. macrocarpon* leaf extract. In accordance with our data, the study’s findings suggest that incorporating *S. macrocarpon* leaf extract into one’s diet may offer protection against SCOP-induced cognitive and neurochemical impairments. Moreover, research suggests that *S. macrocarpon* can protect against mercury-induced neurotoxicity and modulate memory functions, indicating its therapeutic potential in anxiety treatments and memory enhancement [31]. Simultaneously, our data indicated that chronic treatment with SMB may exhibit a protective effect against cholinergic damage (by regulating AChE activity) and oxidative damage (by restoring SOD, CAT, and GPX activity, as well as by attenuating levels of carbonylated proteins and MDA) induced by SCOP in zebrafish. Recent studies have demonstrated that extracts from the leaves of *Solanum macrocarpon* exhibit significant antioxidant activity, protecting cells from oxidative damage, particularly at the hepatic and cerebral levels [54].

The apparent discrepancy between the in silico PASS neurotoxicity predictions (Pa = 0.87–0.88) and the experimentally observed neuroprotective outcomes can be explained by the limitations of the PASS algorithm, which relies exclusively on structural similarity rather than biological context. Redox-active molecules like polyphenols may be misclassified as “potentially neurotoxic” due to their electrophilic centers and ability to interact with redox enzymes, though this very property underlies their adaptive antioxidant function in living systems [45,55].

Therefore, such predictive tools should be interpreted cautiously, as empirical data consistently demonstrate the opposite trend—chlorogenic acid and rutin mitigate oxidative and inflammatory processes, reinforcing their neuroprotective rather than neurotoxic roles [39,40].

This beneficial effect is attributed to the presence of phenolic compounds and flavonoids in plant extracts. Additionally, research has indicated that *Solanum macrocarpon* can inhibit the enzymes AChE and monoamine oxidase, suggesting a neuroprotective potential [28,38].

Regarding the potential toxic/adverse effects of *Solanum macrocarpon*, one study investigated its acute toxicity in Wistar rats using doses of 300 mg/kg and 2000 mg/kg. The results did not indicate significant toxic effects, suggesting that at these doses, the leaves and fruits are safe for consumption [56]. However, other studies have identified the presence of chemical elements such as lead and cadmium in the plant’s composition, which suggests the need for cautious and balanced consumption. Although no major toxic effects are observed at the doses used, it is recommended that its use be diversified and moderate, given the potential risks associated with the presence of these toxic elements [57].

It should also be noted that GAL, used as the reference drug, displayed partial and inconsistent behavioral improvements. This inconsistency may result from interspecies variability in receptor subtypes, differences in pharmacokinetics between zebrafish and mammals, and the single low-dose protocol (1 mg/L) selected to minimize cholinergic overstimulation. Moreover, GAL primarily enhances cholinergic signaling, while cognitive paradigms such as the Y-maze and novel object recognition also rely on dopaminergic and glutamatergic modulation. Consequently, its limited effects in certain tests align with previous zebrafish data showing dose-dependent and test-specific responses [58]. This highlights the broader, multi-target profile of SMB, which integrates antioxidant, anti-inflammatory, and cholinergic mechanisms, resulting in a more comprehensive neuroprotective outcome.

In the current work, chlorogenic acid was detected in very high amount in SMB and rutin content was also moderately high, which is consistent with an earlier data on *Solanum macrocarpon* from Spain indicating chlorogenic acid as the major phenolic acid [59].

Our present findings extend beyond our earlier work on the crude ethanolic extract of *Solanum macrocarpon* [39] by focusing specifically on the *n*-butanol fraction. Unlike the crude extract, which contains a broad mixture of polar and non-polar constituents, SMB concentrates phenolics and flavonoids that appear to underlie its strong antioxidant and anti-cholinesterase effects. Furthermore, in this study, we combined in vivo behavioral assays with in silico ADMET predictions and correlation analyses between biochemical and behavioral markers, thereby providing novel mechanistic insights. These aspects distinguish the present work from our previously published study, despite some overlap in the behavioral and biochemical assays necessary for mechanistic validation.

Both chlorogenic acid and rutin are well-documented for their strong antioxidant properties, contributing significantly to the overall antioxidant capacity of SMB. Similar compounds, including kaempferol-3-rutinoside, were identified in *Solanum macrocarpon* leaf phenolic extract as reported by Salawu et al. [38]. Their study also demonstrated that the extract exhibited considerable DPPH scavenging and ferric reducing antioxidant power. Similarly, a previous study by Wang et al. [60] reported that SMB exhibits strong DPPH radical scavenging activity (84.9%) and a high total phenolic content (34.9 mgGAE/g), further supporting the potent antioxidant capacity of SMB.

These findings suggest that *Solanum macrocarpon* may exert neuroprotective effects through modulation of cholinergic signaling and oxidative stress and supporting cholinergic functions, but further investigations are needed to validate these effects and explore the mechanisms involved.

## 4. Materials and Methods

### 4.1. Retrieval of Molecular Formulas and Chemical Structures

For each compound analyzed, structural information was retrieved from the PubChem database (https://pubchem.ncbi.nlm.nih.gov, accessed on the 20 July 2025), including the molecular formula, the SMILES (Simplified Molecular Input Line Entry System) notation, and the two-dimensional (2D) chemical structure. The data were collected individually, with validation of the consistency between the SMILES code and the corresponding chemical structure, (Table 9) comprising the compound name, molecular formula, SMILES representation, and 2D structural image, which was subsequently used in further analysis stages, including ADMET property prediction and evaluation of the compounds’ biological potential.

### 4.2. In Silico Evaluation of Pharmacokinetic Properties of Major Constituents

To characterize the selected compounds—chlorogenic acid (C_16_H_18_O_9_), rutin (C_25_H_26_O_15_), *p*-coumaric acid (C_9_H_8_O_3_), caffeic acid (C_9_H_8_O_4_), and resveratrol (C_14_H_12_O_3_)—their SMILES notations and molecular formulas were retrieved from the PubChem database (https://pubchem.ncbi.nlm.nih.gov, accessed on the 20 July 2025) and subsequently validated using the RDKit cheminformatics library for Python (https://www.rdkit.org, accessed on the 20 July 2025). The ADMET (Absorption, Distribution, Metabolism, Excretion, and Toxicity) properties were then predicted using the pKCSM platform (https://biosig.lab.uq.edu.au/pkcsm/, accessed on 20 July 2025) [61], SwissADME (http://www.swissadme.ch/, accessed on the 20 July 2025) [62] and (ADMET-AI (https://admet.ai.greenstonebio.com/, accessed on the 20 July 2025) [63], which incorporates Chemprop-RDKit graph neural networks and a reference dataset consisting of 2579 approved drugs available in the DrugBank database (https://go.drugbank.com, accessed on 20 July 2025).

Essential parameters relevant to pharmacokinetic and general toxicological evaluation were assessed, including molecular weight, the partition coefficient (logP), the number of hydrogen bond donors and acceptors, topological polar surface area (TPSA), oral bioavailability, and aqueous solubility. In the context of neuropharmacological research, several critical descriptors were specifically considered: BBB permeability, indicating the compound’s ability to cross the BBB and reach the CNS; hERG channel inhibition, a key safety marker evaluating the potential for cardiac toxicity through blockade of the human ether-à-go-go-related gene (hERG) potassium channel—critical in avoiding adverse cardiac effects that could compromise neuroactive drug profiles; P-glycoprotein inhibition, which influences drug transport and clearance at the CNS level; and Clinical toxicity, estimating the likelihood that a compound lacks toxic effects, a crucial aspect in the safety evaluation of neuropharmacological candidates [39,49].

All values were expressed both as absolute measurements and as comparative percentiles relative to the DrugBank reference set, providing a contextualized overview of each compound’s pharmacological profile.

### 4.3. In Silico Approach for the Evaluation of Neuropharmacological and Toxicological Properties Using the PASS Online Platform

To evaluate the neuropharmacological and toxicological profiles of the selected phenolic compounds, chlorogenic acid, caffeic acid, *p*-coumaric acid, rutin, and resveratrol, in silico predictions were performed using the PASS Online platform (Prediction of Activity Spectra for Substances, available at https://www.way2drug.com/PASSOnline/, accessed on the 21 July 2025) [64]. The chemical structures of the compounds were input in SMILES format, and the PASS Online platform generated probabilities of activity (Pa) and inactivity (Pi) across a broad spectrum of pharmacological and toxicological endpoints. Particular attention was given to properties relevant to neuropharmacological studies, such as antioxidant activity, inhibition of oxidoreductases and lipid peroxidation, modulation of G-protein-coupled receptors (GPCRs), potential therapeutic effects in dementia and macular degeneration, mGluR5 glutamate receptor agonism, as well as predicted neurotoxicity and dependence liability. Data interpretation followed the standard PASS criteria, where compounds were considered potentially active if Pa > 0.5 and Pi < Pa, ensuring reliable prediction accuracy. A comparative analysis was conducted to identify the most promising candidates with therapeutic potential for neurodegenerative disorders and to assess associated toxicological risks.

### 4.4. Plant Material and Extraction

Fresh *S. macrocarpon* leaves were procured from a local market in Oyo, Nigeria, in July 2022. Prof. Omokafe Ugbogu identified the plant material and subsequently authenticated it at the Forest Herbarium Ibadan (FHI), International Institute of Tropical Agriculture (Ibadan, Nigeria), where a voucher specimen was deposited (FHI 113641). The leaves were separated from the stems, immediately frozen, and later ground into a fine powder. The powdered material was macerated in 96% ethanol for 72 h at room temperature. The resulting extracts were filtered and concentrated using a rotary evaporator to obtain the crude ethanol extract of *Solanum macrocarpon* (SMEE). SMEE was reconstituted in a methanol–water mixture (9:1, *v*/*v*) and successively partitioned with aliquots of *n*-hexane, dichloromethane, ethyl acetate, and *n*-butanol. Preliminary phytochemical screening of the fractions indicated that polar constituents such as phenolics, flavonoids, tannins, and saponins were predominantly concentrated in the *n*-butanol fraction (SMB), whereas the n-hexane and dichloromethane fractions were richer in non-polar constituents (lipids, sterols), and the ethyl acetate fraction contained intermediate levels of phenolics and alkaloids. Since flavonoids, alkaloids, saponins, and tannins are the major bioactive constituents implicated in the neuroprotective activity of *Solanum macrocarpon* [12], the *n*-butanol fraction was selected for detailed chemical and biological analysis.

### 4.5. High-Performance Liquid Chromatography

Phenolic and flavonoid compounds in the SMB were determined and quantified using reverse-phase high-performance liquid chromatography (RP-HPLC) on an ACE 5 C18 column (150 × 4.6 mm, 5 μm particle size) at 25 °C, following the method described in our recent works [39,65]. The analysis was performed using an Agilent 1260 Infinity II LC system (Agilent Technologies, Waldbronn, Germany) equipped with a system controller, a quaternary LC pump (G1311B), and a diode array detector (DAD, G7115A).

Detection was primarily conducted at 260 nm, with additional wavelengths set at 280, 320, and 350 nm to enhance compound identification. A total of 23 authentic standards were used for HPLC analysis, including chlorogenic acid, caffeic acid, p-coumaric acid, ferulic acid, gallic acid, catechin, epicatechin, rutin, quercetin, kaempferol, kaempferol-3-rutinoside, luteolin, apigenin, myricetin, resveratrol, syringic acid, vanillic acid, protocatechuic acid, cinnamic acid, naringenin, hesperidin, ellagic acid, and isoquercitrin. These were selected based on prior phytochemical reports on *Solanum macrocarpon* and related Solanaceae species. The mobile phase consisted of solvent A: 0.1% formic acid in 80% acetonitrile, and solvent B: 0.1% formic acid in deionized water. The gradient elution program was as follows: 0–10 min: 5% to 15% A at 0.8 mL/min; 10–15 min: 15% A with flow rate decreasing from 0.8 to 0.6 mL/min; 15–17 min: 15% A at 0.6 mL/min; 17–22 min: 15% to 20% A at 0.8 mL/min; 22–26 min: 20% to 30% A at 0.8 mL/min; 26–34 min: 30% to 100% A at 0.8 mL/min; 34–37 min: 100% A at 1.0 mL/min; 37–42 min: return to initial conditions (5% A). An injection volume of 20 μL was used for each sample. We acknowledge that HPLC-DAD-MS would provide higher accuracy for metabolite verification and plan to include this approach in future studies.

### 4.6. Study Design and Animal Care

A total of 100 adult wild-type zebrafish (*Danio rerio*), aged 5–7 months and exhibiting short fins, were used in this study, with an equal male-to-female ratio (1:1). The average body length ranged from 3 to 4 cm. Fish were purchased from the European Zebrafish Resource Center (Institute of Toxicology and Genetics, Germany) and acclimated under laboratory conditions during a two-week quarantine period in a 70 L tank. The water, disinfected and renewed every two days, was maintained at a temperature of 27 ± 1 °C. Water quality parameters were monitored daily and maintained within the following ranges: pH 7.0–7.5, dissolved oxygen 8 ± 1 mg/L, conductivity 1400–1500 µS/cm, and ammonia/nitrites < 0.001 mg/L. The photoperiod was set to a 14:10 h light–dark cycle. Fish were fed Norwin Norvital flakes (Norwin, Gadstrup, Denmark), using an automatic feeder three times per day (8:00 a.m., 2:00 p.m., and 8:00 p.m.), ensuring full consumption within 10 min.

All experimental procedures were conducted following the ARRIVE guidelines [66] and approved by the Animal Ethics Committee of the Faculty of Biology, Alexandru Ioan Cuza University of Iasi, Romania (Project Approval No. 1714/6 July 2023). The study complied fully with Directive 2010/63/EU of the European Parliament and of the Council on the protection of animals used for scientific purposes. No signs of toxicity or mortality were observed in any of the animals throughout the experimental period.

**Figure 10:**
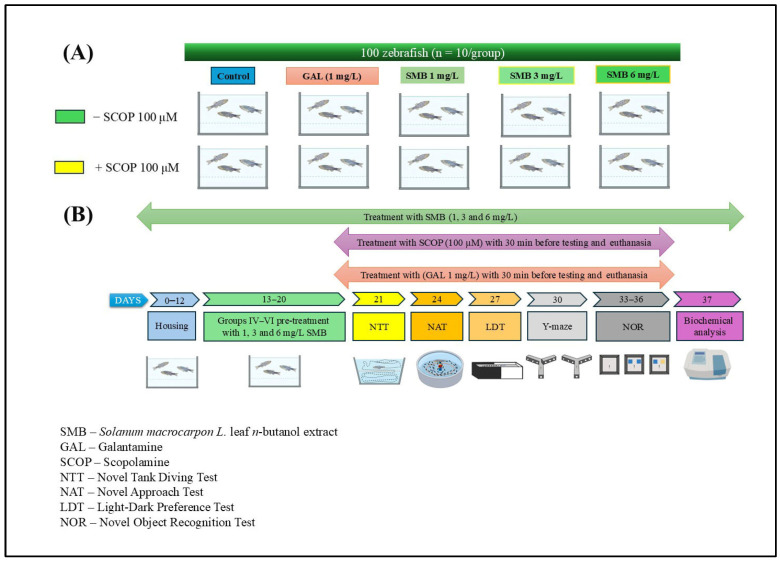
Schematic representation of the experimental protocol. (**A**) Experimental group allocation and treatment design; (**B**) Timeline of behavioral and biochemical assessments.

The zebrafish were randomly divided into 10 experimental groups (n = 10 per group), based on exposure to SCOP (100 μM) (Sigma Aldrich, Darmstadt, Germany) or no SCOP treatment (Figure 10A). Sample size determination was conducted using InVivoStat 4.7, an R-based statistical software package [67]. Based on a significance level (*p*) of 0.05, a sample size of n = 10 zebrafish per group provided a statistical power of 98% to detect a biologically relevant effect size of 20%. This confirmed the adequacy of the group size for detecting meaningful behavioral and biochemical differences. The groups were organized as follows: (I) control group; (II) group exposed to acute GAL (1 mg/L) action, used as a positive standard for behavioral and biochemical analyses; three subgroups (III, IV, and V) treated exclusively with SMB at concentrations of 1, 3, and 6 mg/L; (VI) group treated with SCOP (100 μM); (VII) group exposed concurrently to SCOP and GAL, and three additional subgroups (VIII, IX, and X) receiving acute SCOP treatment and chronic SMB (1, 3, and 6 mg/L) treatment.

GAL was administered via immersion for 3 min before behavioral testing or euthanasia (Group II). In co-treated Group VII, GAL was given immediately after SCOP exposure and just before behavioral tests/euthanasia. SMB was administered chronically by dissolving it in 1% dimethyl sulfoxide (DMSO) and adding it to tank water at the specified concentrations for Groups III–V and VIII–X. SCOP-induced cognitive impairment was established in Groups VI–X by exposing fish for 30 min before behavioral testing, based on validated protocols from previous studies [31,49] (Figure 10B). The chosen dosages for GAL and SMB were informed by literature precedent [49]. The selection of SMB concentrations (1, 3, and 6 mg/L) was based on our previous study conducted in the same zebrafish model, in which the ethanolic extract of *Solanum macrocarpon* leaves (SMEE), administered by immersion at 1, 3, and 6 mg/L, induced dose-dependent neuroprotective and anxiolytic effects [39]. To ensure inter-study comparability and consistency in dose translation between the ethanolic extract (SMEE) and the n-butanolic fraction (SMB), we maintained the same concentration range, which was subsequently confirmed in pilot tests (good tolerability, no mortality, and no nonspecific locomotor effects).

### 4.7. Behavioral Assessments

Locomotor, memory, and anxiety-related behaviors of zebrafish were recorded during in vivo trials using a Logitech C922 Pro HD Stream camera (Logitech, Lausanne, Switzerland). The video recordings were subsequently analyzed using ANY-maze software, version 7.48 (Stoelting Co., Wood Dale, IL, USA), which allowed for precise quantification of movement patterns and behavioral metrics.

#### 4.7.1. Novel Tank Diving Test (NTT)

The NTT was used to assess zebrafish anxiety-like behavior in response to a novel environment. The protocol was adapted from the methodology described by Cachat et al. [68]. The test was conducted in a trapezoidal acrylic tank filled with 1.5 L of water, maintained at 27 ± 1 °C. The tank dimensions were as follows: base length 23.9 cm, top length 28.9 cm, height 15.1 cm, diagonal side 15.9 cm, top width 7.4 cm, and bottom width 6.1 cm. Each fish was individually placed into the tank, and behavior was recorded for 6 min. The tank was virtually divided into two horizontal zones: the top and bottom halves. After testing each group, the tank water was replaced with clean, temperature-matched water to avoid cross-contamination. Behavioral parameters analyzed included: latency to enter the top zone, total time spent in the top and bottom zones, and total distance traveled. These metrics were used to evaluate anxiety-like responses and general locomotor activity.

#### 4.7.2. Novel Approach Test (NAT)

The NAT was used to evaluate zebrafish responses to a novel object, providing insights into anxiety-like and exploratory behaviors [69]. The test was conducted in an opaque white plastic cylindrical arena with a diameter of 34 cm and a height of 15 cm. The tank was filled with water from the fish housing system, maintained at 27 ± 1 °C, and replaced after each set of trials to ensure consistency. The test arena was virtually divided into two zones: an inner zone (a central circle with a 10 cm diameter), where the novel object was placed, and an outer zone (thigmotaxis zone) near the tank wall, typically associated with anxiety-related behavior. Each fish was individually placed in the arena and observed for 5 min. A multicolored Lego figure (5 cm in height), used as the novel object stimulus, was placed at the center of the tank, as described in previous studies [49]. Behavioral endpoints included: time spent in each zone (inner vs. outer), total distance traveled, immobility duration, and latency to approach the novel object. These parameters were used to assess anxiety, neophobia, and exploratory drive in zebrafish.

#### 4.7.3. Light–Dark Transition Test (LDT)

The LDT was employed to assess anxiety-like and exploratory behavior in zebrafish, leveraging their innate preference for darker environments while measuring their willingness to explore brightly lit areas. The test followed the protocol described by Facciol et al. [70]. The experimental setup consisted of a rectangular acrylic tank (55 cm length × 9.5 cm height × 9.5 cm width), divided into two equal sections: a white compartment (light zone) and a black compartment (dark zone). The tank was placed in a uniformly white testing environment to minimize external visual stimuli and reduce environmental bias. Water temperature was maintained at 27 ± 1 °C, and fresh water was introduced after testing each experimental group. Each fish was individually placed at the center of the tank, and its behavior was recorded for 5 min. The following behavioral parameters were analyzed: time spent in the light and dark zones, number of transitions between zones, and total distance traveled. To control potential side bias, the tank was rotated 180° after every seventh trial.

#### 4.7.4. The Novel Object Recognition (NOR)

The NOR test is a widely established paradigm for assessing recognition memory in zebrafish, based on their innate preference for exploring novel stimuli [71]. The procedure consisted of three main phases: habituation, training, and testing. During the habituation, zebrafish were individually placed in a 30 × 30 × 30 cm tank filled with 5 cm of water, devoid of any objects, for 5 min per day over three consecutive days. On the fourth day, the training phase was conducted by exposing the fish to two identical objects placed in the tank for a 10 min session. After a 1 h retention interval, the test phase began, in which one of the familiar objects was replaced with a novel object of similar size and shape but differing in color and texture. Zebrafish behavior was recorded for an additional 10 min. Throughout the experiment, water temperature was maintained at 27 ± 1 °C, and water was replaced between test sessions to ensure uniform conditions across groups.

Recognition memory was evaluated by computing the preference index, calculated as follows:

$$Preference\left(\%\right)=\frac{Time\;spent\;exploring\;the\;NO}{Time\;spent\;exploring\;the\;FO+Time\;spent\;exploring\;the\;NO}\times 100\%$$

A higher preference index indicates a stronger inclination toward the novel object, reflecting intact recognition memory.

### 4.8. Analysis of Biochemical Parameters

Following the completion of behavioral assessments, zebrafish were euthanized using rapid cooling-an accepted physical method involving immersion in ice-cold water (2–4 °C) for 10 min [72]. Immediately after euthanasia, brains were carefully dissected and transferred to pre-chilled tubes.

Tissue homogenization was carried out using a Mikro-Dismembrator U (Sartorius, New York, NY, USA) fitted with 3 mm diameter magnetic beads (Sartorius Stedim Biotech GmbH, Goettingen, Germany). Each brain was homogenized in 0.1 M potassium phosphate buffer (pH 7.4; Chemical Company, Iasi, Romania) supplemented with 1.15% KCl (Chemical Company, Romania) to maintain isotonic conditions.

The resulting homogenates were centrifuged at 960× *g* for 15 min at 4 °C. The supernatant was collected and stored on ice for subsequent enzymatic activity assays and oxidative stress biomarker analysis.

#### 4.8.1. Acetylcholinesterase (AChE) Activity Assay

AChE activity in zebrafish brain homogenates was measured using Ellman’s spectrophotometric method [73]. The assay was based on the enzymatic hydrolysis of acetylthiocholine iodide (ATCh; Sigma Aldrich, Darmstadt, Germany) in the presence of 5,5′-dithio-bis-(2-nitrobenzoic acid) (DTNB; Sigma Aldrich, Darmstadt, Germany) as the chromogenic reagent. The reaction was carried out in phosphate buffer (0.1 M, pH 7.4). During the reaction, thiocholine-produced by the hydrolysis of ATCh-reacts with DTNB to form a yellow-colored 5-thio-2-nitrobenzoate anion, which was quantified by measuring absorbance at 412 nm using a UV-Vis spectrophotometer. AChE activity was expressed as nmol of ATCh hydrolyzed per minute per milligram of protein. Protein concentrations in the samples were determined using the Bradford assay [74].

#### 4.8.2. Superoxide Dismutase (SOD) Activity Assay

SOD activity was determined based on its ability to inhibit the photochemical reduction of nitroblue tetrazolium (NBT; AppliChem, Darmstadt, Germany) in the presence of riboflavin (Sigma Aldrich, Germany), following the protocol described by Artenie [75]. The reaction mixture consisted of 0.067 M potassium phosphate buffer (pH 7.4), enzyme extract, 0.1 M EDTA (Carl Roth, Karlsruhe, Germany), 0.12 mM riboflavin, and 1.5 mM NBT. Following light exposure, the reduction of NBT was monitored spectrophotometrically at 560 nm. One unit of SOD activity was defined as the amount of enzyme required to inhibit NBT reduction by 50%. Enzyme activity was normalized to total protein content and expressed as units per milligram of protein.

#### 4.8.3. Catalase (CAT) Activity Assay

CAT activity was determined following the method of Sinha [76], which is based on the rate of decomposition of hydrogen peroxide (H_2_O_2_, Chemical Company, Iasi, Romania). The reaction mixture consisted of 125 µL of enzyme homogenate and 125 µL of 0.16 M H_2_O_2_ prepared in 0.1 M potassium phosphate buffer (pH 7.4) containing 1.15% KCl (prepared from KH_2_PO_4_ and K_2_HPO_4_; Chemical Company, Iasi, Romania). After 180 s of incubation, the reaction was terminated by adding 500 µL of a potassium dichromate–glacial acetic acid reagent, composed of 5% potassium dichromate (Chemical Company, Romania) and glacial acetic acid (Sigma Aldrich, Darmstadt, Germany). Samples were then incubated at 95 °C for 10 min to develop the chromophore. Following incubation, the samples were centrifuged at 14,000 rpm for 5 min, and the absorbance of the supernatant was measured at 570 nm using a UV-Vis spectrophotometer. CAT activity was expressed as µmol of H_2_O_2_ decomposed per min per milligram of protein.

#### 4.8.4. Glutathione Peroxidase (GPX) Activity Assay

GPX activity was assessed according to the method described by Fukuzawa and Tokumura [77]. The reaction mixture included enzyme extract, 0.25 M phosphate buffer, 25 mM EDTA, and 0.4 M sodium azide (NaN_3_, Sigma Aldrich, Darmstadt, Germany). After 10 min of incubation at 37 °C, reduced glutathione (GSH, Sigma Aldrich, Darmstadt, Germany) and H_2_O_2_ were added, and the reaction continued for another 10 min. The reaction was terminated with metaphosphoric acid (Sigma Aldrich, Darmstadt, Germany), and absorbance was measured at 412 nm after centrifugation and reagent addition. Enzyme activity was expressed in units *per* mg of protein.

#### 4.8.5. Carbonylated Protein Content Assay

The quantification of protein carbonylation was performed using the 2,4-dinitrophenylhydrazine (DNPH, Sigma Aldrich, Darmstadt, Germany), derivatization method described by Luo et al. [78]. This assay is based on the reaction between DNPH and oxidized protein residues, leading to the formation of 2,4-dinitrophenylhydrazones, which were measured spectrophotometrically at 370 nm. The results were normalized to protein concentration and expressed as nmol DNPH/mg of protein.

#### 4.8.6. Malondialdehyde (MDA) Level Assay

Lipid peroxidation was assessed by quantifying MDA levels using the thiobarbituric acid reactive substances (TBARS) assay, following the method of Ohkawa et al. [79]. The reaction mixture contained 200 µL of brain homogenate, 1 mL of 50% trichloroacetic acid (TCA, Chimreactiv, Bucharest, Romania), 1 mL of 26 mM thiobarbituric acid (Sigma Aldrich, Darmstadt, Germany), and 0.1 M HCl (Chemical Company, Iasi, Romania). The mixture was vortexed and heated at 95 °C for 20 min, followed by rapid cooling on ice for 5 min. Samples were centrifuged at 960× *g* for 10 min, and the absorbance of the supernatant was measured at 532 nm. The MDA content was expressed as nmol/mg of protein.

All biochemical measurements were conducted in triplicate to ensure data reliability, and appropriate blank and calibration curves were used for each assay to enhance accuracy and reproducibility.

### 4.9. Data Analysis

All results were expressed as mean ± standard error of the mean (S.E.M.). Comparison between groups was performed using one-way analysis of variance (ANOVA), followed by Tukey’s post hoc test to identify significant differences based on treatment. The statistical significance threshold was set at *p* < 0.05. Statistical analyses were conducted using GraphPad Prism 9.4 software (GraphPad Software, Inc., San Diego, CA, USA). Additionally, correlations between behavioral parameters, enzymatic activities, and lipid peroxidation levels were evaluated using the Pearson correlation coefficient (*r*) to explore potential relationships between the investigated variables.

## 5. Conclusions

In conclusion, the administration of SMB demonstrated a significant impact on ameliorating SCOP-induced behavioral deficits in zebrafish, by reducing anxiety-like behaviors and improving cognitive functions. The treatment, especially at concentrations of 3 and 6 mg/mL, regulated AChE activity and had a notable antioxidant effect, restoring the enzymatic activity of SOD, CAT, and GPX, as well as reducing levels of MDA and carbonylated proteins. These effects suggest a significant neuroprotective mechanism. These findings indicate the need for further investigations to clarify the molecular mechanisms and evaluate the potential risks associated with the use of this plant.

## Figures and Tables

**Table 1:** Amount of chlorogenic acid and rutin in *Solanum macrocarpon* L. leaf *n*-butanol extract (SMB) is calculated from standard curves, retention times, and linear relationships between peak areas and concentrations.

Sample	Compounds	Retention Time (min)	Maximum Absorbance (nm)	Amount (mg/g, Mean ± SD)	Standard Curve	R^2^
SMB	Chlorogenic acid	15.595	320	162.39 ± 0.27	y = 28.919x + 54.92	0.9974
	Rutin	30.000	260	24.61 ± 0.56	y = 36.127x + 150.42	0.9993

Notably, kaempferol-3-rutinoside, which has been reported as a major flavonoid in *Solanum macrocarpon* leaves [38], was not detected in our SMB sample. This discrepancy may reflect differences in geographic origin, growth conditions, or extraction/fractionation methods. The absence of this compound under our conditions highlights the importance of evaluating phytochemical variability across different preparations.

**Table 2:** Pharmacokinetic and drug-likeness properties of the analysed compounds.

Property	Chlorogenic Acid	Rutin
Molecular weight (Da)	354.1	610.52
Log P	–0.65	–1.69
TPSA (Å^2^)	164.75	268.43
HBA (H-bond Acceptors)	8	16
HBD (H-bond Donors)	6	10
Lipinski Rule Compliance	3/4	1/4
QED (Drug-likeness Score)	0.23	0.14
Stereocenters	4	10

**Table 3:** ADMET Absorption Parameters of the Studied Compounds.

Parameters	Chlorogenic Acid	Rutin
Human intestinal absorption	0.88	0.09
Oral bioavailability	0.29	0.18
Aqueous solubility (log mol/L)	–1.21	–3.86
Lipophilicity (logP)	–1.83	0.77
Hydration free energy (kcal/mol)	–16.41	–15.67
Cell effective permeability (log cm/s)	–6.60	–6.82
PAMPA permeability	0.07	0.09
P-glycoprotein inhibition	0.01	0.14

**Table 4:** Distribution parameters of the studied compounds in comparison with DrugBank percentiles.

Parameters	Chlorogenic Acid	Rutin
BBB permeability	0.40 (24.35%)	0.06 (3.02%)
PPB (%)	59.12 (30.90%)	84.88 (63.32%)
Vdss (L/kg)	2.03 (54.36%)	6.39 (81.74%)

**Table 5:** Metabolic parameters of the studied compounds.

Parameters	Chlorogenic Acid	Rutin
CYP1A2 Inhibition	0.04 (49.71%)	0.01 (34.74%)
CYP2C19 Inhibition	0.04 (34.20%)	0.03 (29.78%)
CYP2C9 Inhibition	0.02 (43.23%)	0.02 (36.91%)
CYP2D6 Inhibition	0.03 (42.26%)	0.03 (42.73%)
CYP3A4 Inhibition	0.003 (24.47%)	0.01 (33.11%)
CYP2C9 Substrate	0.18 (56.69%)	0.03 (9.42%)
CYP2D6 Substrate	0.02 (17.29%)	0.02 (12.95%)
CYP3A4 Substrate	0.22 (25.28%)	0.41 (42.50%)

**Table 6:** Excretion parameters of the studied compounds.

Parameters	Chlorogenic Acid	Rutin
Half Life	0.00 (16.98%) h	49.51 (87.05%) h
Drug Clearance (Hepatocyte)	22.94 (35.67%) µL/min/10^6^ cells	25.57 (38.66%) µL/min/10^6^ cells
Drug Clearance (Microsome)	0.00 (22.53%) µL/min/mg	40.10 (70.84%) µL/min/mg

**Table 7:** Predicted toxicity parameters of the studied compounds.

Parameters	Chlorogenic Acid	Rutin
hERG blocking	0.06 (23.26%)	0.65 (69.99%)
Clinical toxicity	0.17 (63.28%)	0.20 (65.99%)
Mutagenicity	0.14 (42.88%)	0.60 (88.02%)
Drug-induced liver injury	0.48 (52.62%)	0.75 (67.74%)
Carcinogenicity	0.02 (7.91%)	0.02 (10.62%)
Acute toxicity LD50	1.99 (19.39%)	2.90 (73.40%)
Skin reaction	0.28 (32.11%)	0.21 (22.64%)
Androgen receptor (full length)	0.12 (88.02%)	0.08 (83.68%)
Androgen receptor (ligand binding domain)	0.08 (88.68%)	0.12 (91.47%)
Aryl hydrocarbon receptor	0.03 (53.16%)	0.11 (73.09%)
aromatase	0.02 (49.09%)	0.14 (79.80%)
Estrogen receptor (full length)	0.11 (58.94%)	0.29 (88.06%)
Estrogen receptor (ligand binding domain)	0.07 (84.30%)	0.21 (93.87%)
Peroxisome proliferator-activated receptor gamma	0.02 (72.12%)	0.01 (58.08%)
Nuclear factor (erythroid-derived 2)-like 2/antioxidant responsive element	0.17 (57.31%)	0.18 (58.67%)
ATPase family AAA domain-containing protein 5 (ATAD5)	0.03 (72.51%)	0.07 (85.03%)
Heat shock factor response element	0.02 (54.28%)	0.02 (55.29%)
Mitochondrial membrane potential	0.03 (46.53%)	0.12 (66.23%)
Tumor protein p53	0.06 (67.47%)	0.22 (87.09%)

**Table 8:** Predicted neuropharmacological and toxicological activities of phenolic compounds from *Solanum macrocarpon* L. leaf n-butanol extract (SMB), based on PASS online analysis.

Parameters	Chlorogenic Acid		Rutin	
	Pa	Pi	Pa	Pi
Antioxidant	0.785	0.004	0.923	0.003
Oxidoreductase inhibitor	0.846	0.004	0.694	0.016
Lipid peroxidase inhibitor	0.855	0.003	0.987	0.001
G-protein-coupled receptor kinase inhibitor	0.716	0.021	0.257	0.184
Dementia treatment	0.258	0.209	0.541	0.008
Age-related macular degeneration treatment	0.210	0.161	-	-
Glutamate (mGluR5) agonist	0.133	0.106	-	-
Dopamine precursors	0.041	0.015	0.033	0.026
NADH-kinase inhibitor	0.199	0.108	-	-
Neurotoxic	0.874	0.008	0.882	0.007
Dependence	0.257	0.183	-	-

**Table 9:** Summary of structural features of selected phenolic compounds: compound names, molecular formulas, SMILES representations, and 2D chemical structures.

Compound and Molecular Formula	2D Structure	SMILES
Chlorogenic acid C_16_H_18_O_9_	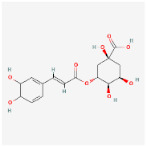	C1[C@H]([C@H]([C@@H](C[C@@]1(C(=O)O)O) OC(=O)/C=C/C2=CC(=C(C=C2)O)O)O)O
Rutin C_25_H_26_O_15_	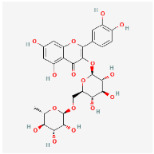	C[C@H]1[C@@H]([C@H]([C@H]([C@@H](O1) OC[C@@H]2[C@H]([C@@H]([C@H]([C@@H] (O2)OC3=C(OC4=CC(=CC(=C4C3=O)O)O)C5=CC (=C(C=C5)O)O)O)O)O)O)O)O
p-Coumaric acid C_9_H_8_O_3_	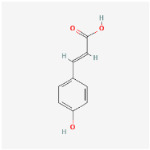	C1=CC(=CC=C1/C=C/C(=O)O)O
Caffeic acid C_9_H_8_O_4_	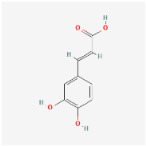	C1=CC(=C(C=C1/C=C/C(=O)O)O)O
Resveratrol C_14_H_12_O_3_	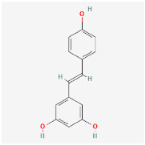	C1=CC(=CC=C1/C=C/C2=CC(=CC(=C2)O)O)O

## Data Availability

The data presented in this study are available on request from the corresponding author. The data are not publicly available due to privacy and ethical restrictions.

## References

[1] Pritam P., Deka R., Bhardwaj A., Srivastava R., Kumar D., Jha A.K., Jha N.K., Villa C., Jha S.K. (2022). Antioxidants in Alzheimer’s Disease: Current Therapeutic Significance and Future Prospects. Biology.

[2] Bai R., Guo J., Ye X.Y., Xie Y., Xie T. (2022). Oxidative Stress: The Core Pathogenesis and Mechanism of Alzheimer’s Disease. Ageing Res. Rev..

[3] Fronza M.G., Alves D., Praticò D., Savegnago L. (2023). The Neurobiology and Therapeutic Potential of Multi-Targeting β-Secretase, Glycogen Synthase Kinase 3β and Acetylcholinesterase in Alzheimer’s Disease. Ageing Res. Rev..

[4] Gajendra K., Pratap G.K., Poornima D.V., Shantaram M., Ranjita G. (2024). Natural Acetylcholinesterase Inhibitors: A Multi-Targeted Therapeutic Potential in Alzheimer’s Disease. Eur. J. Med. Chem. Rep..

[5] Majdi A., Sadigh-Eteghad S., Aghsan S.R., Farajdokht F., Vatandoust S.M., Namvaran A., Mahmoudi J. (2020). Amyloid-β, Tau, and the Cholinergic System in Alzheimer’s Disease: Seeking Direction in a Tangle of Clues. Rev. Neurosci..

[6] Kepp K.P., Robakis N.K., Høilund-Carlsen P.F., Sensi S.L., Vissel B. (2023). The Amyloid Cascade Hypothesis: An Updated Critical Review. Brain.

[7] Rawat K., Tewari D., Bisht A., Chandra S., Tiruneh Y.K., Hassan H.M., Al-Emam A., Sindi E.R., Al-Dies A.A.M. (2024). Identification of AChE Targeted Therapeutic Compounds for Alzheimer’s Disease: An in-Silico Study with DFT Integration. Sci. Rep..

[8] Buccellato F.R., D’Anca M., Galimberti D., Fenoglio C., Scarpini E. (2021). Role of Oxidative Damage in Alzheimer’s Disease and Neurodegeneration: From Pathogenic Mechanisms to Biomarker Discovery. Antioxidants.

[9] Roy R.G., Mandal P.K., Maroon J.C. (2023). Oxidative Stress Occurs Prior to Amyloid Aβ Plaque Formation and Tau Phosphorylation in Alzheimer’s Disease: Role of Glutathione and Metal Ions. ACS Chem. Neurosci..

[10] Wang R.P.H., Huang J., Chan K.W.Y., Leung W.K., Goto T., Ho Y.S., Chang R.C.C. (2023). IL-1β and TNF-α Play an Important Role in Modulating the Risk of Periodontitis and Alzheimer’s Disease. J. Neuroinflamm..

[11] Plantone D., Pardini M., Righi D., Manco C., Colombo B.M., De Stefano N. (2023). The Role of TNF-α in Alzheimer’s Disease: A Narrative Review. Cells.

[12] Nafea M., Elharoun M., Abd-Alhaseeb M.M., Helmy M.W. (2023). Leflunomide Abrogates Neuroinflammatory Changes in a Rat Model of Alzheimer’s Disease: The Role of TNF-α/NF-ΚB/IL-1β Axis Inhibition. Naunyn Schmiedebergs Arch. Pharmacol..

[13] Walczak-Nowicka Ł.J., Herbet M. (2021). Acetylcholinesterase Inhibitors in the Treatment of Neurodegenerative Diseases and the Role of Acetylcholinesterase in Their Pathogenesis. Int. J. Mol. Sci..

[14] Knopman D.S., Amieva H., Petersen R.C., Chételat G., Holtzman D.M., Hyman B.T., Nixon R.A., Jones D.T. (2021). Alzheimer Disease. Nat. Rev. Dis. Primers.

[15] Sharma C., Kim S.R., Barone E., Trostchansky A. (2021). Linking Oxidative Stress and Proteinopathy in Alzheimer’s Disease. Antioxidants.

[16] Rahman M.A., Rahman M.H., Biswas P., Hossain M.S., Islam R., Hannan M.A., Uddin M.J., Rhim H. (2020). Potential Therapeutic Role of Phytochemicals to Mitigate Mitochondrial Dysfunctions in Alzheimer’s Disease. Antioxidants.

[17] Bhattacharya R.S., Singh R., Panghal A., Thakur A., Singh L., Verma R.K., Singh C., Goyal M., Kumar J. (2025). Multi-Targeting Phytochemicals for Alzheimer’s Disease. Phytother. Res..

[18] Bakrim S., Aboulaghras S., El Menyiy N., El Omari N., Assaggaf H., Lee L.H., Montesano D., Gallo M., Zengin G., AlDhaheri Y. (2022). Phytochemical Compounds and Nanoparticles as Phytochemical Delivery Systems for Alzheimer’s Disease Management. Molecules.

[19] Calfio C., Gonzalez A., Singh S.K., Rojo L.E., MacCioni R.B. (2020). The Emerging Role of Nutraceuticals and Phytochemicals in the Prevention and Treatment of Alzheimer’s Disease. J. Alzheimer’s Dis..

[20] Maqbool Z., Arshad M.S., Ali A., Aziz A., Khalid W., Afzal M.F., Bangar S.P., Addi M., Hano C., Lorenzo J.M. (2022). Potential Role of Phytochemical Extract from Saffron in Development of Functional Foods and Protection of Brain-Related Disorders. Oxid. Med. Cell Longev..

[21] Nematzadeh G., Abdulhamid Abdulkareem D., Abdulhamid Abdulkareem K., Bello A., Abdul N., Olatunji Sidiq K., Umar Olayinka B., Kareem I., Muazu Danzaki M., Toyin Mustapha O. (2024). Phylogenetic Analysis of *Solanum macrocarpon*: The Evolutionary Relationships and Species Diversification. J. Plant Mol. Breed..

[22] Kayode J., Janet Ayeni M. (2020). Growth, Yield, Nutritional and Mineral Composition of *Solanum macrocarpon* L. as Affected by Fertilizer Application. J. Biotechnol. Res..

[23] Ogidi O.I., Ukamaka E., Ogidi O.I., Chukwudi P., Ibe A.I., Eze P.U., Canus T.N., History A. (2021). Preliminary phytochemical profile and antimicrobial potentials of white-green african garden egg (*Solanum macrocarpon*) fruits obtained from Yenagoa. ASIO J. Pharm. Herb. Med. Res..

[24] Famuwagun A.A., Taiwo K.A., Gbadamosi S.O., Oyedele D.J., Aluko R.E., Adebooye O.C. (2017). Extraction Optimization and Antioxidant Properties of African Eggplant (*Solanum macrocarpon*) Leaf Polyphenols. J. Food Qual..

[25] Anjorin O.J., Karigidi K.O., Agunloye M.O., Olaiya C.O. (2023). Antidiabetic Effects of Fractions of Methanol Extract of *Solanum macrocarpon* Linn. On Streptozotocin—Induced Diabetic Male Wistar Rats. Vegetos.

[26] Adewale O., Onasanya A., Fadaka A., Iwere H., Anadozie S., Osukoya O., Olayide I. (2014). In Vitro Antioxidant Effect of Aqueous Extract of *Solanum macrocarpon* Leaves in Rat Liver and Brain. Oxid. Antioxid. Med. Sci..

[27] Ajiboye B.O., Akalabu M.C., Ojo O.A., Afolabi O.B., Okesola M.A., Olayide I., Oyinloye B.E. (2018). Inhibitory Effect of Ethyl Acetate Fraction of *Solanum macrocarpon* L. Leaves on Cholinergic, Monoaminergic, and Purinergic Enzyme Activities. J. Food Biochem..

[28] Ogunsuyi O.B., Ademiluyi A.O., Oboh G. (2020). Solanum Leaves Extracts Exhibit Antioxidant Properties and Inhibit Monoamine Oxidase and Acetylcholinesterase Activities (In Vitro) in Drosophila Melanogaster. J. Basic Clin. Physiol. Pharmacol..

[29] Idowu G.P., Obuotor E.M., Onajobi F.D. (2021). In Vitro and in Silico Investigation of Cholinesterase Inhibition and Anti-Radical Properties of *Solanum macrocarpon* Leaf Extracts: A Preliminary Anti-Alzheimer’s Study. Alzheimer’s Dement..

[30] Ogunsuyi O.B., Omage F.B., Olagoke O.C., Oboh G., Rocha J.B.T. (2023). Phytochemicals from African Eggplants (*Solanum macrocarpon* L.) and Black Nightshade (*Solanum nigrum* L.) Leaves as Acetylcholinesterase Inhibitors: An in-Silico Study. J. Biomol. Struct. Dyn..

[31] Ogunsuyi O.B., Olagoke O.C., Afolabi B.A., Loreto J.S., Ademiluyi A.O., Aschner M., Oboh G., Barbosa N.V., da Rocha J.B.T. (2022). Effect of Solanum Vegetables on Memory Index, Redox Status, and Expressions of Critical Neural Genes in Drosophila Melanogaster Model of Memory Impairment. Metab. Brain Dis..

[32] Agunloye O.M., Ogunsuyi O.B., Oluokun O.O., Oboh G. (2024). Amnesiac (AMN) Gene and Cnc/Nrf2-Redox Responses in Fruit Fly Model of Memory Impairment Co-Administered Solanum Leaves and Donepezil. Pharmacol. Res.-Mod. Chin. Med..

[33] Okesola M.A., Ajiboye B.O., Oyinloye B.E., Osukoya O.A., Owero-ozeze O.S., Ekakitie L.I., Kappo A.P. (2020). Effect of *Solanum macrocarpon* Linn Leaf Aqueous Extract on the Brain of an Alloxan-Induced Rat Model of Diabetes. J. Int. Med. Res..

[34] Shenoy A., Banerjee M., Upadhya A., Bagwe-Parab S., Kaur G. (2022). The Brilliance of the Zebrafish Model: Perception on Behavior and Alzheimer’s Disease. Front. Behav. Neurosci..

[35] Boiangiu R.S., Mihasan M., Gorgan D.L., Stache B.A., Hritcu L. (2021). Anxiolytic, Promnesic, Anti-Acetylcholinesterase and Antioxidant Effects of Cotinine and 6-Hydroxy-L-Nicotine in Scopolamine-Induced Zebrafish (Danio Rerio) Model of Alzheimer’s Disease. Antioxidants.

[36] Rathi K.M., Undale V.R., Wavhale R.D., Mohammed F.S., Karwa P.N., Patil H. (2025). From Computational Screening to Zebrafish Testing: Repurposing of Doxazosin, Donepezil, and Dolutegravir for Neuroprotective Potential in Alzheimer’s Disease. Naunyn Schmiedebergs Arch. Pharmacol..

[37] Sande R., Godad A., Doshi G. (2024). Zebrafish Experimental Animal Models for AD: A Comprehensive Review. Curr. Rev. Clin. Exp. Pharmacol..

[38] Salawu S., Akindahunsi A., Ibukun E., Duodu K. (2013). Antioxidant Activities and Inhibitory Action of *Solanum macrocarpon* and *Hibiscus esculentus* Phenolic Containing Leaf Extracts against Lipid Oxidation. Int. J. Med. Plants Res..

[39] Brinza I., Guliev C., Oresanya I.O., Gok H.N., Orhan I.E., Hritcu L. (2025). *Solanum macrocarpon* L. Ethanolic Leaf Extract Exhibits Neuroprotective and Anxiolytic Effects in Scopolamine-Induced Amnesic Zebrafish Model. Pharmaceuticals.

[40] Popovici L.-F., Brinza I., Oancea S., Hritcu L. (2025). Chronic Administration of Calendula Officinalis Ethanolic Extract Mitigates Anxiety-like Behavior and Cognitive Impairment Induced by Acute Scopolamine Exposure in Zebrafish. Pharmaceuticals.

[41] Coradini K., de Andrade D.F., Altenhofen S., Reolon G.K., Nery L.R., Silva N.E., Vianna M.R.M.R., Bonan C.D., Beck R.C.R. (2021). Free and Nanoencapsulated Curcumin Prevents Scopolamine-Induced Cognitive Impairment in Adult Zebrafish. J. Drug Deliv. Sci. Technol..

[42] Fu C.W., Tong S.K., Liu M.X., Liao B.K., Chou M.Y. (2025). Scopolamine Affects Fear Learning and Social Recognition in Adult Zebrafish. Neuroscience.

[43] Karunakaran K.B., Thiyagaraj A., Santhakumar K. (2022). Novel Insights on Acetylcholinesterase Inhibition by Convolvulus Pluricaulis, Scopolamine and Their Combination in Zebrafish. Nat. Prod. Bioprospect..

[44] Figueira I., Menezes R., Macedo D., Costa I., Nunes C., Santos D. (2017). Polyphenols Beyond Barriers: A Glimpse into the Brain. Curr. Neuropharmacol..

[45] Scuto M., Rampulla F., Reali G.M., Spanò S.M., Trovato Salinaro A., Calabrese V. (2024). Hormetic Nutrition and Redox Regulation in Gut–Brain Axis Disorders. Antioxidants.

[46] Magalingam K.B., Radhakrishnan A., Haleagrahara N. (2016). Protective Effects of Quercetin Glycosides, Rutin, and Isoquercetrin against 6-Hydroxydopamine (6-OHDA)-Induced Neurotoxicity in Rat Pheochromocytoma (PC-12) Cells. Int. J. Immunopathol. Pharmacol..

[47] Dogra A. (2024). Phytotherapeutic Potential of Rutin Against Xenobiotic-Induced Toxicities in Preclinical Models. Food Rev. Int..

[48] Suraweera T.L., Vasantha Rupasinghe H.P., Dellaire G., Xu Z. (2020). Regulation of Nrf2/ARE Pathway by Dietary Flavonoids: A Friend or Foe for Cancer Management?. Antioxidants.

[49] Popovici L.F., Brinza I., Gatea F., Badea G.I., Vamanu E., Oancea S., Hritcu L. (2025). Enhancement of Cognitive Benefits and Anti-Anxiety Effects of Phytolacca Americana Fruits in a Zebrafish (Danio Rerio) Model of Scopolamine-Induced Memory Impairment. Antioxidants.

[50] Osakabe N., Anfuso C.D., Jacob U.M., Sidenkova A., Fritsch T., Abdelhameed A.S., Rashan L., Wenzel U., Calabrese E.J., Calabrese V. (2024). Phytochemicals and Vitagenes for a Healthy Brain. Brain and Mental Health in Ageing. Healthy Ageing and Longevity.

[51] Sahebnasagh A., Eghbali S., Saghafi F., Sureda A., Avan R. (2022). Neurohormetic Phytochemicals in the Pathogenesis of Neurodegenerative Diseases. Immun. Ageing.

[52] Mary O.O., Sebastine O.U., Ejuiwa M.C., Ikemefuna O.I., Dominic E. (2020). Anxiolytic and Curative Effect of *Solanum macrocarpon* Leaves Extract on Acetaminophen Induced Brain Injury in Adult Wistar Rats. J. Pharmacogn. Phytochem..

[53] Ogunsuyi O.B., Ademiluyi A.O., Oboh G., Oyeleye S.I., Dada A.F. (2018). Green Leafy Vegetables from Two Solanum Spp. (*Solanum nigrum* L. and *Solanum macrocarpon* L.) Ameliorate Scopolamine-Induced Cognitive and Neurochemical Impairments in Rats. Food Sci. Nutr..

[54] Usunobun U., Samuel E.I. (2016). Phytochemical Analysis, Mineral Composition and in Vitro Antioxidant Activities of Celosia Argentea Leaves. Int. J. Sci. World.

[55] Kaur R., Sood A., Lang D.K., Bhatia S., Al-Harrasi A., Aleya L., Behl T. (2022). Potential of Flavonoids as Anti-Alzheimer’s Agents: Bench to Bedside. Environ. Sci. Pollut. Res..

[56] Dougnon V., Bankolé H., Edorh P., Klotoé J.R., Dougnon J., Fah L., Loko F., Boko M. (2013). Acute Toxicity of *Solanum macrocarpon* Linn (Solanaceae) on Wistar Rats: Study about Leaves and Fruits. Am. J. Biochem..

[57] Dougnon T.V., Bankolé H.S., Johnson R.C., Klotoé J.R., Dougnon G., Gbaguidi F., Assogba F., Gbénou J., Sahidou S., Atègbo J.-M. (2012). Phytochemical Screening, Nutritional and Toxicological Analyses of Leaves and Fruits of *Solanum macrocarpon* Linn (Solanaceae) in Cotonou (Benin). Food Nutr. Sci..

[58] Aryal S., Skinner T., Bridges B., Weber J.T. (2020). The Pathology of Parkinson’s Disease and Potential Benefit of Dietary Polyphenols. Molecules.

[59] Plazas M., Prohens J., Cuñat A.N., Vilanova S., Gramazio P., Herraiz F.J., Andújar I. (2014). Reducing Capacity, Chlorogenic Acid Content and Biological Activity in a Collection of Scarlet (*Solanum aethiopicum*) and Gboma (*S. macrocarpon*) Eggplants. Int. J. Mol. Sci..

[60] Wang D., Huang J., Wang H., Oresanya I.O., Orhan I.E., Heil J., Morlock G.E. (2024). African Under-Utilized Medicinal Leafy Vegetables Studied by Microtiter Plate Assays and High-Performance Thin-Layer Chromatography–Planar Assays. Molecules.

[61] Pires D.E.V., Blundell T.L., Ascher D.B. (2015). PkCSM: Predicting Small-Molecule Pharmacokinetic and Toxicity Properties Using Graph-Based Signatures. J. Med. Chem..

[62] Daina A., Michielin O., Zoete V. (2017). SwissADME: A Free Web Tool to Evaluate Pharmacokinetics, Drug-Likeness and Medicinal Chemistry Friendliness of Small Molecules. Sci. Rep..

[63] Swanson K., Walther P., Leitz J., Mukherjee S., Wu J.C., Shivnaraine R.V., Zou J. (2024). ADMET-AI: A Machine Learning ADMET Platform for Evaluation of Large-Scale Chemical Libraries. Bioinformatics.

[64] Way2Drug—Main. https://www.way2drug.com/PASSOnline/index.php.

[65] Oresanya I.O., Gök H.N., Akkol E.K., Sonibare M.A., Orhan I.E. (2025). Phenolic Acids in Some Under-Utilized Medicinal and Leafy Vegetables, Their Anti-Inflammatory and Wound Healing Activities. S. Afr. J. Bot..

[66] du Sert N.P., Hurst V., Ahluwalia A., Alam S., Avey M.T., Baker M., Browne W.J., Clark A., Cuthill I.C., Dirnagl U. (2020). The ARRIVE Guidelines 2.0: Updated Guidelines for Reporting Animal Research. PLoS Biol..

[67] Bate S.T., Clark R.A. (2014). The Design and Statistical Analysis of Animal Experiments.

[68] Cachat J.M., Canavello P.R., Elkhayat S.I., Bartels B.K., Hart P.C., Elegante M.F., Beeson E.C., Laffoon A.L., Haymore W.A.M., Tien D.H. (2011). Video-Aided Analysis of Zebrafish Locomotion and Anxiety-Related Behavioral Responses. Neuromethods.

[69] Hamilton T.J., Morrill A., Lucas K., Gallup J., Harris M., Healey M., Pitman T., Schalomon M., Digweed S., Tresguerres M. (2017). Establishing Zebrafish as a Model to Study the Anxiolytic Effects of Scopolamine. Sci. Rep..

[70] Facciol A., Iqbal M., Eada A., Tran S., Gerlai R. (2019). The Light-Dark Task in Zebrafish Confuses Two Distinct Factors: Interaction between Background Shade and Illumination Level Preference. Pharmacol. Biochem. Behav..

[71] Faillace M., Pisera-Fuster A., Medrano M., Bejarano A., Bernabeu R. (2017). Short- and Long-Term Effects of Nicotine and the Histone Deacetylase Inhibitor Phenylbutyrate on Novel Object Recognition in Zebrafish. Psychopharmacology.

[72] Gupta T., Mullins M.C. (2010). Dissection of Organs from the Adult Zebrafish. JoVE (J. Vis. Exp.).

[73] Ellman G.L., Courtney K.D., Andres V., Feather-Stone R.M. (1961). A New and Rapid Colorimetric Determination of Acetylcholinesterase Activity. Biochem. Pharmacol..

[74] Bradford M.M. (1976). A Rapid and Sensitive Method for the Quantitation of Microgram Quantities of Protein Utilizing the Principle of Protein-Dye Binding. Anal. Biochem..

[75] Artenie V., Ungureanu E., Negură A.M. (2008). Metode de Investigare a Metabolismului Glucidic Și Lipidic.

[76] Sinha A.K. (1972). Colorimetric Assay of Catalase. Anal. Biochem..

[77] Fukuzawa K., Tokumura A. (1976). Glutathione Peroxidase Activity in Tissues of Vitamin E-Deficient Mice. J. Nutr. Sci. Vitaminol..

[78] Luo S., Wehr N.B. (2009). Protein Carbonylation: Avoiding Pitfalls in the 2,4-Dinitrophenylhydrazine Assay. Redox Rep..

[79] Ohkawa H., Ohishi N., Yagi K. (1979). Assay for Lipid Peroxides in Animal Tissues by Thiobarbituric Acid Reaction. Anal. Biochem..

